# The Short-Term Value of the “Healthy Primary School of the Future” Initiative: A Social Return on Investment Analysis

**DOI:** 10.3389/fpubh.2020.00401

**Published:** 2020-08-21

**Authors:** Marije Oosterhoff, Onno C. P. van Schayck, Nina H. M. Bartelink, Hans Bosma, Maartje Willeboordse, Bjorn Winkens, Manuela A. Joore

**Affiliations:** ^1^Department of Clinical Epidemiology and Medical Technology Assessment, Maastricht University Medical Centre, Care and Public Health Research Institute, Maastricht University, Maastricht, Netherlands; ^2^Department of Family Medicine, Care and Public Health Research Institute, Maastricht University, Maastricht, Netherlands; ^3^Department of Health Promotion, Care and Public Health Research Institute, Maastricht University, Maastricht, Netherlands; ^4^Department of Social Medicine, Care and Public Health Research Institute, Maastricht University, Maastricht, Netherlands; ^5^Department of Methodology and Statistics, Care and Public Health Research Institute, Maastricht University, Maastricht, Netherlands

**Keywords:** social return on investment, health promotion/economics, child, health promoting schools, economic evaluation

## Abstract

**Background:** This study examines the social return on investment (SROI) of the “Healthy Primary School of the Future” initiative after 2 years.

**Methods:** Healthy Primary Schools of the Future (HPSF) provide a healthy lunch and daily structured physical activity sessions, whereas Physical Activity Schools (PAS) focus on physical activity only. We evaluated the 2-years investments and effects (*N* = 1,676 children) of both school environments (four schools) compared to control schools (four schools). Investments and outcomes were grouped within the healthcare, education, household & leisure, and labor & social security sector. Outcomes that could be expressed in monetary terms were used for the calculation of social return on investment.

**Results:** HPSF and PAS created outcomes for the healthcare sector by favorable changes in health behaviors, body mass index [both significant], and medical resource use [not significant]. Outcomes for the education sector included a favorable impact on perceived social behaviors and school satisfaction, and absenteeism from school [latter not significant], and more engagement with the community was experienced. The per child investments, €859 (HPSF) and €1017 (PAS), generated a benefit of €8 (HPSF) and €49 (PAS) due to reduced school absenteeism and medical resource use.

**Conclusions:** Within 2 years of intervention implementation, the HPSF initiative created outcomes in several sectors, but the benefits did not outweigh the investments. Follow-up assessments as well as modeling long-term outcomes are needed to assess the total value of the interventions. Until then, the SROI framework can inform strategies for obtaining stakeholder support and intervention implementation.

**Trial registration:** The study was registered in the ClinicalTrials.gov database on 14 June 2016 (NCT02800616).

## Introduction

The environment in which today's children are growing up is characterized by many opportunities for unhealthy dietary intake and few facilities for physical activity (1, 2). Adverse consequences, such as overweight and obesity have been steadily increasing over the last decades. In 2009, 13–15% of Dutch boys and girls aged 2–21 years were overweight compared to 5–7% in 1980 (3). As a response to this growing public health concern, the Healthy Primary School of the Future initiative was developed (4). Key elements of this initiative are the provision of a daily healthy lunch and structured physical activity sessions, which are innovative elements within the Dutch primary school setting (corresponding to 4–12 years of age). The HPSF initiative consists of a full intervention, named the “Healthy Primary Schools of the Future” (HPSF), and a partial intervention, referred to as “Physical Activity Schools” (PAS). Within 2 years of intervention, Bartelink et al. (14) found that HPSF was effective in increasing healthy dietary behaviors and physical activity (5). Both HPSF and PAS were also effective in lowering children's body mass index (BMI) z-scores (BMI adjusted for age and sex) (6).

Cost-effectiveness studies aim to inform implementation and funding decisions. Ideally, the time over which costs and outcomes of childhood programs are evaluated should go beyond childhood, because the impact of weight reductions on chronic diseases, health-related quality of life (HRQOL), and costs do not fully occur within childhood. Evaluations of short-term cost and effects are, however, more in line with the time horizons that policy makers tend to work with (usually 3–5 years) (7, 8), and provide information for decision-making on the implementation, continuation, and scaling-up of interventions.

In the current study, we use the social return on investment (SROI) framework for examining the investments, outcomes, and societal value of HPSF and PAS. The SROI framework aims to examine all outcomes of a program (no matter who incurs them). To aggregate investments and outcomes and calculate return on investment or cost-effectiveness, outcomes are assigned to a monetary value (financial benefits) and are divided by the investments. This calculation (SROI calculation) results in an estimate on the amount of benefits returned for every euro spent (9, 10). The SROI framework also recognizes that not all outcomes can be assigned to a monetary value (and can be expressed quantitatively). Outcomes that cannot be expressed in monetary terms are included in a SROI story, which articulates the non-monetary value. The objective of this study is to examine the short-term SROI generated by HPSF and PAS in the first 2 years of intervention implementation.

## Methods

A quasi-experimental study, which started in 2015 in the south of the Netherlands, evaluates the effects of two “Healthy Primary Schools of the Future” (HPSF) and two “Physical Activity Schools” (PAS) compared to four control schools who maintained the usual school curriculum. No randomization was applied because voluntary participation was key to the intervention implementation. A healthy morning snack and daily healthy lunches were provided (at HPSF only) in combination with structured physical activity sessions including structured sports activities, free play, and creative activities. At HPSF, the lunch break was prolonged to about 1 h, which led to an extension of the school day with ~30 min (some lunch breaks involved an educational component to meet the education hour requirements). Children and their parents were invited to participate in data collection at baseline (no blinding), and could join at all measurement waves as children continuously leave and enter primary school (dynamic cohort design). Further details on the interventions and data collection procedures have been published elsewhere (4, 11).

A SROI analysis was performed by taking five steps, according to the methods of Nicholls et al. (2012): (1) defining the scope and identifying key stakeholders; (2) identifying investments and outcomes; (3) evidencing outcomes; (4) establishing impact, and (5) assessing the SROI (12).

### Step 1: Scope and Key Stakeholders

**Figure 1:**
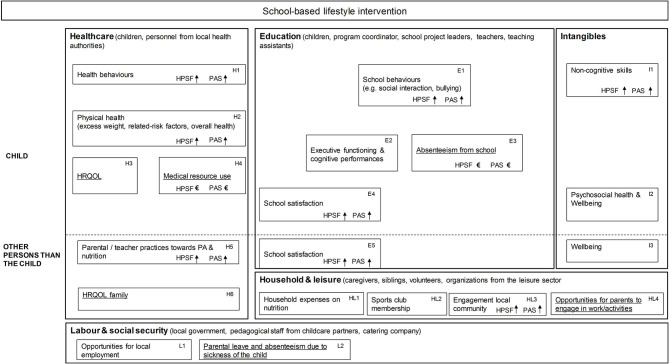
Impact inventory including the potential outcomes of school-based lifestyle interventions. HRQOL, health-related quality of life; PA, physical activity. Underlined text: The outcomes that could be expressed in financial terms (H3, H4, H6, E3, HL4, L2) were included in the calculation of social return on investment. Due to the small numbers for unpaid work (HL4) and parental absenteeism from work or education (L2), the potential benefits could not be reliably estimated and we refrained from including them in the calculation of social return on investment. We refrained from defining financial proxies for outcomes that are not financial in nature (e.g., behaviors) as this will lead to double counting due to the interdependencies between outcomes within the same or between domains (e.g., health behaviors, physical health, and HRQOL; between physical health and absenteeism from school). Non-financial outcomes, however, may have some additional value. We therefore complemented the SROI calculation with a SROI story. A longer time horizon was needed to examine the outcomes on cognitive performances (E2). HL1, HL2, and L1 were not (yet) formally measured within the quasi-experimental study.

Whilst the quasi-experimental study examines the effects of HPSF and PAS for a period of 4 years (baseline: school year 2015/2016, year 4: 2019/2020), the current study focuses on the impact after 2 years (baseline: school year 2015/2016, year 1: 2016/2017, year 2: 2017/2018). Several stakeholders contributed to the delivery of HPSF and PAS, and may be directly or indirectly affected by the interventions. Stakeholders were grouped within the healthcare, education, household & leisure, and labor & social security sector (Figure 1: box H, box E, box HL, and box L, respectively).

### Step 2 and 3: Identifying Investments and Outcomes and Evidencing Outcomes

#### Investments

In a previous study, we made an overview of the activities provided at HPSF and PAS in comparison to the regular school curriculum, and the corresponding investment costs (13). The cost analysis also revealed cost offsets: children's lunches were provided at school and led to a cost offset within the household, and the extended school day at HPSF provided caregivers with additional time (productivity cost) that could be spent on paid or unpaid work. These cost offsets were deducted from the investments to calculate the net investments. For more details on the cost calculation see Appendix 2.

#### Qualitative Outcomes

Semi-structured interviews with stakeholders were held at the end of the second year about the implementation and perceived changes (11). School coordinators (*N* = 4), coordinators of the pedagogical employees (*N* = 4), school health promoters from the regional Public Health Services (*N* = 4), and the project coordinator (*N* = 1) were interviewed (Figure 1 and Appendix 3). More information can be found in the paper written by Bartelink et al. (14).

#### Quantitative Outcomes

The selection of quantitative outcomes was made by the interdisciplinary scientific project group, and presented in an impact inventory (Figure 1) (4). Quantitative outcomes were measured annually (T0: 2015, T1: 2016, T2: 2017) and covered height and weight measurements, child questionnaires, and parental questionnaires. School records contained information on absenteeism from school. Routine school satisfaction surveys were administered among caregivers and children between 2016 and 2018. In the current study, we included children that were enrolled at the participating schools and exposed to the interventions from baseline onwards. Children in grade 8 at baseline were excluded as no follow-up measurements could be obtained. We also excluded the children that switched between schools between 2015 and 2017. Benefits were calculated by multiplying the outcomes over 2 years (measured in volumes/quantity) by the unit cost for that outcome. Benefits were assessed at the group level in order to calculate an average per child benefit. Standardized prices from national costing guidelines were used for outcomes that were financial in nature (medical resource use, productivity) (15), and published proxy values were applied for other outcomes (QALY and school absenteeism) (Table A3.1) (16, 17). The outcome measurement and valuation, and the selection of the study sample (*N* = 1,676) are further described in Appendix 3.

### Step 4: Establishing Impact

This step is used to estimate what proportion of the outcome can be isolated as being added by the intervention. In the current study, the quasi-experimental design with a control group and 2-years time frame was used to account for this element.

### Step 5: SROI Assessment

The SROI calculation included the outcomes on children's HRQOL, medical resource use (Figure 1: H3 and H4), and school absenteeism (Figure 1: E3). The outcomes for medical resource use and absenteeism, which represented a cost, were rescaled so that all outcomes would indicate a benefit. Benefits were calculated as the sum of outcomes over year 1 and 2. Firstly, the per child benefits were aggregated within each sector, and were then summed up across the sectors (within-dimension approach) (18). An annual discount rate of 2.5% was used for investments and benefits to account for the differences in the time at which investments and outcomes occur (16). The reasons for in/excluding outcomes from the SROI calculation, and the calculation of benefits are further detailed in Appendix 4.

### Statistical Analysis

Descriptive statistics were used to explore baseline sociodemographic and outcome variables. Multiple imputation was used to account for possible selective non-response (missing at random assumption) and to use all available data (see Appendix 4 for details on the handling of missing data). Imputations were generated with the MICE package in R using 50 imputed datasets with 20 iterations (for details see Appendix 4) using predictive mean matching. The mean differences in the per child benefits over year 1 and 2 were examined with a generalized linear model with a Gamma distribution and a log link function to account for the zero values and skewness of the data. The analyses were adjusted for sex, study year at baseline, socioeconomic status (SES), ethnicity, baseline BMI z-scores (BMI adjusted for age and sex), and baseline outcome scores to account for imbalances in covariates (Appendix 4). The social return on investment was calculated as the ratio of benefits to net investments. Additionally, the incremental net monetary benefit was calculated as the difference in benefits (for HPSF and PAS vs. control schools) minus the difference in net investments. All statistical analyses were performed in IBM SPSS Statistics for Windows version 23 (Armonk, NY: IBM Corp) and R version 3.5.1.

### Scenario and Sensitivity Analysis

Scenario analyses were performed to analyse the SROI of HPSF and PAS for specific situations. For details and reasoning behind the scenario analyses we refer to Appendix 5. (1) Lower investments at HPSF for pedagogical staff (eight instead of 12 pedagogical workers) based on changes in the way activities are organized. (2) Lower investments for HPSF and PAS that are expected to occur on the long-term (so-called steady state) (13). (3) Excluding children in grade 7 at baseline who are leaving school after the eighth grade and missed the 2-years follow-up measurement. Sensitivity analyses were conducted to see how the results would change under different assumptions: (1) Including spillover effects on caregiver's HRQOL and productivity (paid work) (Figure 1: H6, HL4). (2) No offsets due to the extended school day at HPSF in the calculation of the net investment for HPSF. (3 and 4) Willingness to pay (WTP) thresholds of €20.000 and €50.000 per QALY gained instead of €36.000 per QALY gained. (5) No discounting of benefits and investments instead of an annual discount rate of 2.5%. (6) A complete case analysis (non-imputed outcomes). For details on the sensitivity analyses, see also Appendix 5.

## Results

At baseline (T0), *N* = 1,403, 60.3% children and their parents joined the study. For the current study, *N* = 1,676 children and their parents were included based on the selection of school years and school switchers excluded (see Appendix 1 for the flow diagram) (6). Children from control schools had higher BMI z-scores (0.232 vs. 0.051 at HPSF and 0.092 at PAS) and chronic diseases were more prevalent (36 vs. 30% at HPSF and PAS) (Table 1).

**Table 1:** Summary of covariates and baseline outcomes for each group separately (pre-imputation).

**Baseline characteristics (*N* = 1,676)**		**HPSF (*N* = 537)**	**PAS (*N* = 478)**	**Control (*N* = 661)**	**Total (*N* = 1,676)**	**Missing (%)**
Covariates	Gender Boys (*N*, %)	256 (47.7%)	226 (47.3%)	312 (47.2%)	794 (47.4%)	0%
	Grade (mean ± sd)	4.0 ± 2.00	3.8 ± 2.01	4.1 ± 1.99	4.0 ± 2.00	0%
	Age in years (mean ± sd)	7.6 ± 2.16	7.4 ± 2.22	7.6 ± 2.13	7.5 ± 2.16	0%
	Ethnicity (*N*, %)^a^					39.4%
	Native background	273 (80.1%)	283 (86.8%)	285 (81.7%)	841 (82.8%)	
	Western background	44 (12.9%)	30 (9.2%)	41 (11.7%)	115 (11.3%)	
	Non-Western background	24 (7.0%)	13 (4.0%)	23 (6.6%)	60 (5.9%)	
	Education level mother at baseline (*N*, %)					33.7%
	Low	63 (17.6%)	62 (17.0%)	77 (19.8%)	202 (18.2%)	
	Intermediate	171 (47.9%)	181 (49.6%)	178 (45.8%)	530 (47.7%)	
	High	123 (34.5%)	122 (33.4%)	134 (34.4%)	379 (34.1%)	
	Education level father at baseline (*N*, %)					60.4%
	Low	32 (14.2%)	47 (20.2%)	47 (22.9%)	126 (19.0%)	
	Intermediate	110 (48.7%)	107 (45.9%)	83 (40.5%)	300 (45.2%)	
	High	84 (37.2%)	79 (33.9%)	75 (36.6%)	238 (35.8%)	
	Net monthly household income (*N*, %)					
	Up to €1,500	20 (11.8%)	19 (10.6%)	22 (13.3%)	61 (11.8%)	69.2%
	€1,500 to < €2,500	21 (12.4%)	25 (13.9%)	32 (19.3%)	78 (15.1%)	
	€2,500 to < €3,500	64 (37.6%)	70 (38.9%)	53 (31.9%)	187 (36.2%)	
	€3,500 and above	65 (38.2%)	66 (36.7%)	59 (35.5%)	190 (36.8%)	
	Socioeconomic status at baseline (*N*, %)^b^					33.4%
	Low	104 (28.8%)	118 (32.3%)	142 (36.3%)	364 (32.6%)	
	Intermediate	129 (35.7%)	130 (35.6%)	121 (30.9%)	380 (34.0%)	
	High	128 (35.5%)	117 (32.1%)	128 (32.7%)	373 (33.4%)	
	BMI z-score (mean ± sd)	0.051 ± 1.01	0.092 ± 0.95	0.232 ± 1.07	0.135 ± 1.02	33.8%
	Chronic diseases (medication/admission/visit) (*N*, %)	70 (29.5%)	73 (29.9%)	78 (36.1%)	221 (31.7%)	58.4%
HRQOL	Utility of the child (mean ± sd)	0.954 ± 0.10	0.945 ± 0.11	0.946 ± 0.11	0.948 ± 0.11	41.5%
Medical resource use within the last 12 months	GP visits, costs per child (mean ± sd)	21.3 ± 41.04	31.1 ± 54.31	19.3 ± 39.40	24.1 ± 45.95	58.4%
	Speech therapist visits, costs per child (mean ± sd)	45.7 ± 212.35	58.3 ± 238.40	60.0 ± 244.40	54.6 ± 231.65	58.4%
	Specialists visits, costs per child (mean ± sd)	74.4 ± 193.34	77.1 ± 193.26	72.8 ± 270.38	74.8 ± 219.78	58.4%
	Physiotherapist/occupational therapist visits, costs per child (mean ± sd)	13.5 ± 140.03	5.1 ± 43.94	6.0 ± 51.38	8.2 ± 89.63	55.0%
	Youth care visits, costs per child (mean ± sd)	46.4 ± 435.58	4.6 ± 39.90	27.7 ± 187.70	25.8 ± 274.84	58.4%
	Psychologist / social worker visits, costs per child (mean ± sd)	13.8 ± 65.51	23.0 ± 162.12	37.9 ± 256.75	24.5 ± 176.50	58.4%
	Hospital admissions, costs per child (mean ± sd)	103.7 ± 1260.76	106.2 ± 762.09	82.9 ± 588.89	98.1 ± 921.56	58.2%
	Medication, costs per child (mean ± sd)	38.8 ± 180.18	10.8 ± 51.61	9.9 ± 66.33	20.0 ± 115.81	59.0%
	Total healthcare costs (mean ± sd)	360.4 ± 1526.36	318.6 ± 969.27	320.4 ± 961.65	333.3 ± 1183.82	59.2%
HRQOL family	Utility of the primary caregiver (mean ± sd)^c^	0.922 ± 0.17	0.922 ± 0.14	0.917 ± 0.13	0.921 ± 0.15	57.2%
Absenteeism from school	Annual health-related absenteeism days (mean ± sd)	7.25 ± 7.63	8.05 ± 9.96	6.67 ± 8.31	7.29 ± 7.72	44.0%
	Annual other absenteeism days (mean ± sd)	0.86 ± 3.12	0.68 ± 2.05	0.83 ± 3.38	0.79 ± 2.93	44.0%
Parental absenteeism	Any absenteeism (N, %)	8 (3.4%)	4 (1.6%)	2 (0.9%)	14 (2.0%)	58.6%
	Parental absenteeism days from work or education due to health of the child (mean ± sd)	0.08 ± 0.59	0.06 ± 0.54	0.01 ± 0.15	0.05 ± 0.48	
Parental working hours	Working hours/week for paid work (mean ± sd)					
	Total	57.73 ± 16.97	58.90 ± 15.06	57.49 ± 15.90	58.07 ± 15.97	60.5%
	1 caregiver per household	25.44 ± 8.14	25.00 ± 7.84	34.90 ± 3.90	28.81 ± 8.05	
	2 caregivers per household	59.19 ± 15.84	60.10 ± 13.82	58.65 ± 15.41	59.35 ± 15.00	
	Any working hours for unpaid work (N, %)	9 (3.8%)	9 (3.6%)	20 (8.8%)	38 (5.2%)	57.2%
	Working hours/week for unpaid work (mean ± sd)					
	Total	0.396 ± 2.16	0.212 ± 1.36	0.890 ± 4.30	0.49 ± 2.85	
	1 caregiver per household	1.06 ± 2.91	0.70 ± 2.36	1.86 ± 5.04	1.23 ± 3.67	
	2 caregivers per household	0.35 ± 2.10	0.17 ± 1.24	0.79 ± 4.22	0.42 ± 2.76	

*BMI, body mass index; GP, general practitioner; HPSF, Healthy Primary School of the Future; HRQOL, health-related quality of life; PAS, Physical Activity School*.

a *Combined for baseline, T1, and T2 due to static nature of ethnicity*.

b *Average of standardized scores on the education of the mother, education of the father, and income adjusted for household size*.

c *HRQOL measured with the EuroQol-5 Dimensions Questionnaire (EQ5D). This measure evaluates the subjective HRQOL of a person. Health states are then adjusted for the preference of a health state (valuations obtained from the general public), which results in a value between 0 (worst possible health state) and 1 (perfect health)*.

### Investments

For the first year of implementation, the total investments amounted to €1,448 per child for HPSF and €665 per child for PAS. The offsets for HPSF included the forgone household expenses on children's lunches as they were provided by the schools, the value of the extended school day in terms of parental productivity, and the forgone household expenses on the fee for the lunch break (used for supervision during the lunch break) which was not applied (total €−1,019 per child). The net investment costs of HPSF were €429 per child for the first year of intervention implementation (€2.68 per child per day) (not discounted). The offsets for PAS only included the forgone household expenses on the fee for the lunch break (the offsets for the lunch and the prolonged school day were not applicable for PAS), and the net investment amounted to €505 per child for the first year (€3.16 per child per day) (not discounted) (19). In the second year, the costs for transport and accommodations were not incurred, which resulted in a net investment of €399 (HPSF) and €475 (PAS) per child (not discounted). The net investment for year 1 and 2 together were €828 per child for HPSF (€2.59 per child per day) and €980 for PAS (€3.06 per child per day) (discounted results: €859 per child year and €2.69 per child per day for HPSF, and €1,017 per child year and €3.18 per child per day for PAS).

### SROI Story (Non-monetized Outcomes)

#### Child Outcomes

##### Healthcare

Bartelink et al. (2019) previously reported that water consumption, the intake of different food types during the lunch, and time spent in light physical activity had increased more at HPSF compared to control schools (5). Several participants on the interviews reported that dietary behaviors of children at HPSF became more diverse, and children were more willing to taste unfamiliar products (Figure 1: H1) (14). Standardized BMI scores had decreased more in children at HPSF compared to children at PAS and control schools, and for children at PAS in comparison to children at control schools (6) (Figure 1: H2).

##### Education

From the interviews with stakeholders it emerged that children were less bored during recess time and fewer conflicts happened at the schoolyard and in the classroom (Figure 1: E1) (14). The school satisfaction surveys showed that children were satisfied with the lunch (60–83% at the two HPSF schools), and with the PA sessions at HPSF and PAS (75–93%). Nearly half of the children at HPSF enjoyed the school day more (39–50%) compared to the pre-intervention period, compared to 46–57% of children at PAS (Figure 1: E4).

##### Intangibles

Regarding non-cognitive skills, the interview respondents noticed that children learnt from the offered games as children were better able to create and manage their own activities during recess time (Figure 1: I1). No significant differences were found between groups for children's self-efficacy scores (adjusted mean differences HPSF vs. control: −0.48 [95% CI: −1.38; 0.42] PAS vs. control: 0.30 [95% CI: −0.60; 1.19]). Self-reported psychosocial health (Figure 1: I2), measured with the Pediatric Quality of Life (PedsQL) instrument, decreased somewhat from baseline to year 2 (no statistically significant differences between groups). This was, however, not observed for parent-reports about children's psychosocial health (Appendix 6).

#### Outcomes in Other Persons Than the Child

##### Healthcare

Some teacher and parental practices (discussing and educating about nutrition and PA) changed in a favorable direction (Figure 1: H5) (14).

##### Education

Caregivers were generally satisfied with the organization (67–74%) and the content of the lunch (70–76%) at HPSF, and with the structured sports activities, free play, and creative activities at HPSF and PAS (50–79%). The majority would recommend HPSF to other caregivers (64–76%) (Figure 1: E5).

##### Intangibles

No significant changes over time were found between groups for parental well-being (adjusted mean differences HPSF vs. control: 0.28 [95% CI: −0.37; 0.93], PAS vs. control: 0.08 [95% CI: −0.61; 0.76]) (Figure 1: I3).

##### Household and leisure

Organizations for sport and leisure were invited to provide workshops. Activities did not only take place at school, but the school gym and the green area around the school were also used for free play and games (Figure 1: HL3). At year 2, 12–18% of the respondents on the parental questionnaire reported that their own working hours and/or the working hours of their partner changed as a result of HPSF and PAS, varying from minor to a lot of influence.

##### Labor and social security

No statistically significant differences were found for parental absenteeism from work or education (Appendix 6) (Figure 1: L2). As this result was based on only few cases (known for <10% of the analyzed study participants) the benefits could not be reliably estimated, and we refrained from including this benefit in the calculation of social return on investment.

### SROI Calculation (Monetized Outcomes)

No statistically significant differences were found between groups for the number of QALYs accrued by children and for children's medical resource use within the 2-years of follow-up (Figure 1: H3 and H4). No statistically significant differences were found for health-related and non-health related absenteeism (Figure 1: E3). Because absenteeism represents a cost, absenteeism days were represented as a negative benefit. The monetary value (per child per 2 years) for health-related absenteeism amounted to €−309 (PAS) and €−352 (HPSF) vs. €−338 (control schools), and were €−29 (PAS) and €−25 (HPSF) vs. €−31 (control schools) for non-health related absenteeism (Table 2). No significant differences were found between groups for the number of QALYs accrued by caregivers (Figure 1: H6): rate ratio HPSF vs. control: 1.00 [95% CI: 0.97; 1.04], rate ratio PAS vs. control: 1.01 [95% CI: 0.97; 1.04]. No statistically significant differences were found for time spent on paid work (Figure 1: HL4): rate ratio HPSF vs. control: 1.02 [95% CI: 0.97; 1.08], PAS vs. control: 1.05 [95% CI: 0.99; 1.12]. The net investment for HPSF (€859/child/2 years), generated a benefit of €8/child/2 years [95% CI: €−1,085–1,057] when considering the financial outcomes in the child (Table 2). The incremental net benefit of HPSF was estimated at €−851/child/2 years (SROI ratio of 0.01). The net investment costs for PAS (€1017/child/2 years), generated a benefit of €49/child/2 years [95% CI: €−1,041–1,097]. The incremental net benefit of PAS was estimated at €−968/child/2 years (SROI ratio of 0.05). See Appendix 6 for the results when not adjusting for covariates. A breakdown of results by sector shows that most investment were incurred by the education sector, while offsets were received by the household and leisure sector, and most benefits belonged to the healthcare sector (HPSF: 100%, PAS: 40%) (Table 3).

Table 2Social return on investment (€) in year 1 and 2 (*N* = 1,676, adjusted for covariates).

**Table:** 

**Panel A: Net investment**	**HPSF vs. control schools**	**PAS vs. control schools**
	**€ per child** **(discounted results)**	**€ per child** **(discounted results)**
Net investment year 1	440	518
Net investment year 2	420	499
Total social opportunity costs (year 1 and 2)^a^	859	1,017

**Table:** 

**Panel B: Benefits**	**Unit cost**	**Control schools**		**HPSF**		**PAS**		**HPSF vs. control schools**				**PAS vs. control schools**			
								**Rate ratio^b^**		**Benefit**		**Rate ratio^b^**		**Benefit**	
										**€ per child Y1 + Y2^c^**				**€ per child Y1 + Y2^c^**	
		**Mean**	**(SE)**	**Mean**	**(SE)**	**Mean**	**(SE)**	**Estimate**	**(95% CI)**	**Estimate**	**(95% CI)**	**Estimate**	**(95% CI)**	**Estimate**	**(95% CI)**
QALYs child	€36,000/QALY^d^	68,508	(335.0)	68,554	(332.6)	68,531	(319.6)	1.00	(0.98; 1.02)	0	(−1304; 1304)	1.00	(0.98; 1.02)	0	(−1304; 1304)
Medical resource use	See Appendix 2^e^	−1,056	(211.3)	−997	(204.9)	−1,010	(194.4)	0.98	(0.81; 1.18)	20	(−180; 191)	0.98	(0.81; 1.18)	20	(−180; 191)
HR absenteeism	€26.48/day^e, f^	−338	(15.6)	−352	(17.6)	−309	(15.9)	1.05	(0.92; 1.19)	−16	(−61; 26)	0.92	(0.81; 1.06)	26	(−19; 61)
Other school absenteeism	€26.48/day^e, f^	−31	(6.8)	−25	(7.4)	−29	(6.6)	0.85	(0.93; 1.16)	4	(−5; 2)	0.90	(0.63; 1.24)	3	(−7; 11)
Total benefits (total year 1 and year 2)										8	(−1085; 1057)			49	(−1041; 1097)

**Table:** 

**Panel C: Social return on investment**	**HPSF vs. control schools**		**PAS vs. control schools**	
	**€ per child Y1 + Y2**		**€ per child Y1 + Y2**	
	**Estimate (95% CI)**	**(95% CI)**	**Estimate**	**(95% CI)**
Ratio of benefits to investments^g^	0.01	(−1.3; 1.2)	0.05	(−1.0; 1.1)
Net monetary benefit^h^	−851	(−1945; 198)	−968	(−2058; 80)
Net monetary benefit per child per day ^i^	−2.66	(−6.08; 0.62)	−3.03	(−6.43; 0.25)

*The analyses were adjusted for sex, study year at baseline, socioeconomic status (SES), ethnicity, baseline BMI z-scores, and baseline outcome scores*.

a *Net investment = investments minus delivery-related offsets (HPSF: household expenses on lunches for children, and the value of the extended school day for parental productivity. HPSF & PAS: forgone household expenses regarding the fee for the lunch break)*.

b *Ratio of mean benefits for HPSF or PAS vs. control schools*.

c Benefits of HPSF or PAS = mean value at control schools * rate ratio (repeated for lower and upper bound of the confidence interval). Discounted with an annual discount rate of 2.5% to account for differential timing of investments and benefits.

d *Pomp et al. (16)*.

e *Because medical resource use and school absenteeism represent a cost, they are represented as a negative benefit*.

f *Guideline for intersectoral costs and benefits of preventive interventions (OCW kerncijfers 2007–2011) (16)*.

g *Ratio of total of benefits and net investments*.

h *Incremental net monetary benefit = incremental benefits – incremental net investments*.

i *For a total of 160 schooldays per year (total of 320 days for 2 years)*.

*CI, confidence interval; HPSF, Healthy Primary School of the Future; HR, health-related; IQR, interquartile range; PAS, Physical Activity School; QALYs, quality-adjusted life years; SE, standard error*.

Table 3Breakdown of social return on investment (€) in year 1 and 2 by sector.

**Table:** 

	**HPSF vs. control schools**			**PAS vs. control schools**		
	**Healthcare sector**	**Education sector**	**Household & leisure sector**	**Healthcare sector**	**Education sector**	**Household & leisure sector**
**Panel A: Net investments**	**€ per child (discounted results)**	**€ per child (discounted results)**	**€ per child (discounted results)**	**€ per child (discounted results)**	**€ per child (discounted results)**	**€ per child (discounted results)**
Net investment year 1	0	1,248	−999	0	663	−146
Net investment year 2	0	1,433	−1,014	0	649	−149
Total social opportunity costs (year 1 and 2)^a^	0	2,862	−2,002	0	1,312	−295

**Table:** 

**Panel B: Benefits (see Table 2)**	**€ per child Y1 + Y2^b^**		**€ per child Y1 + Y2^b^**		**€ per child Y1 + Y2^b^**		**€ per child Y1 + Y2^b^**		**€ per child Y1 + Y2^b^**		**€ per child Y1 + Y2^b^**	
	**Estimate**	**(95% CI)**	**Estimate**	**(95% CI)**	**Estimate**	**(95% CI)**	**Estimate**	**(95% CI)**	**Estimate**	**(95% CI)**	**Estimate**	
QALYs child	0	(−1304; 1304)	NA		NA		0	(−1304; 1304)	NA		NA	
Medical resource use^c^	20	(−180; 191)	NA		NA		20	(−180; 191)	NA		NA	
HR absenteeism^c^	NA		−16	(−61; 26)	NA		NA		26	(−19; 61)	NA	
Other school absenteeism^c^	NA		4	(−5; 2)	NA		NA		3	(−7; 11)	NA	
Total benefits (total year 1 and 2)	20	(−1113; 1123)	−12	(−66; 28)	0	(0; 0)	20	(−1113; 1123)	29	(−26; 72)	0	(0; 0)
**Panel C: Social return on investment**	**€ per child Y1 + Y2**		**€ per child Y1 + Y2**		**€ per child Y1 + Y2**		**€ per child Y1 + Y2**		**€ per child Y1 + Y2**		**€ per child Y1 + Y2**	
	**Estimate**	**(95% CI)**	**Estimate**	**(95% CI)**	**Estimate**	**(95% CI)**	**Estimate**	**(95% CI)**	**Estimate**	**(95% CI)**	**Estimate**	
Ratio of benefits to investments^d^	NA		−0.00	(−1.00; 0.01)	NA		NA		0.02	(−0.02; 0.06)	NA	
Net monetary benefit^e^	20	(−1113; 1123)	−2873	(−5723; −2834)	2002	(NA)	20	(−1113; 1123)	−1283	(−2595; −1240)	295	(NA)
Net monetary benefit per child per day^f^	0.06	(−3.48; 3.51)	−8.98	(−17.89; −8.86)	6.26	(NA)	0.06	(−3.48; 3.51)	−4.01	(−8.11; −3.87)	0.92	(NA)

a *Net investments = investments minus delivery-related offsets (HPSF: household expenses on lunches for children, and the value of the extended school day for parental productivity. HPSF & PAS: forgone household expenses regarding the fee for the lunch break)*.

b Benefits of HPSF or PAS = mean value at control schools * rate ratio (repeated for lower and upper bound of the confidence interval). Discounted with an annual discount rate of 2.5% to account for differential timing of investments and benefits.

c *Because medical resource use and school absenteeism represent a cost, they are represented as a negative benefit*.

d *Ratio of total of benefits and net investments*.

e *Incremental net monetary benefit = incremental benefits - incremental net investments*.

f *For a total of 160 schooldays per year (total of 320 days for two years)*.

*CI, confidence interval; HPSF, Healthy Primary School of the Future; HR, health-related; IQR, interquartile range; PAS, Physical Activity School; QALYs, quality-adjusted life years; SE, standard error*.

The results of the scenario and sensitivity analyses were comparable to the base-case (SROI between zero and one), except for excluding children who were in grade 7 at baseline, and for the complete case analysis (Appendix 7). Repeating the analysis without children in grade 7 resulted in extra benefit for both HPSF and PAS (SROI HPSF: 1.70 vs. 0.01; PAS: 0.70 vs. 0.05). Additionally, including spillovers on caregiver's HRQOL and productivity increased the SROI (SROI HPSF: 0.05 vs. 0.01, PAS: 0.70 vs. 0.05). The complete case analysis showed comparable results with regard to the direction of the regression estimates (Appendix 5). However, due to the number of missing data and the missing data mechanism (not completely at random) the complete case analysis resulted in inefficient and (probably) biased point estimates of the benefits (SROI HPSF: −1.80 vs. 0.01, PAS: 0.60 vs. 0.05).

## Discussion

The objective of the current study was to examine the short-term return on investment created by HPSF and PAS after 2 years of intervention. HPSF and PAS led to outcomes within the healthcare sector (favorable changes in health behaviors and body mass index [both significant], and medical resource use [not significant]), education sector (favorable changes on perceived social behaviors at school and school satisfaction, and absenteeism from school [later not significant]), and household & leisure sector (perceived engagement with the community). The benefits (HPSF: €0.05 per child/day, PAS: €0.31/child/day), did not outweigh the net investment costs of HPSF and PAS (HPSF: €2.69/child/day, PAS: €3.18/child/day). For every euro invested, HPSF and PAS generated a benefit of €0.01 [95% CI: €−1.3; €1.2] and €0.05 [95% CI: €−1.0; €1.1], respectively. In the paper of Bartelink et al. (2019) it was shown that HPSF resulted in more favorable effects on children's BMI scores compared to PAS (6). The authors suggested that HPSF may be more effective in targeting health behaviors, since HPSF simultaneously addressed nutrition and physical activity, and the activities at HPSF led to additional health-promoting changes in the school (5). In contrast, the SROI calculation revealed that PAS led to more financial benefits than HPSF, which was mainly due to the favorable effects of PAS on absenteeism from school (not statistically significant). The results, however, do not suggest that PAS had a more favorable SROI as compared to HPSF, because HPSF led to more favorable results on the non-monetized outcomes (see SROI story). The findings on financial benefits need to be interpreted with caution. Benefits were not statistically significant and therefore uncertain, but a trend toward favorable outcomes was observed. The SROI of HPSF and PAS increased substantially after including spillover effects, which was driven by the monetization of the relative small effects on caregivers' HRQOL (WTP for a QALY), as well as by the favorable effects on caregiver productivity. In the sensitivity analysis, it can also be seen that the SROI was sensitive to the QALY gains. Most short-term cost-effectiveness studies on childhood lifestyle interventions examined cost-effectiveness by the ratio of costs and health outcomes, such as body mass index improvements, cases of overweight prevented, or units of waist circumference prevented (20). WTP thresholds are not available for these outcomes, and interpreting their cost-effectiveness results therefore remains difficult. In contrast to these studies, we combined a qualitative and quantitative approach for examining the health- and non-health outcomes of HPSF and PAS. By using the SROI framework we were able to integrate the outcomes for multiple sectors (21, 22). If we would have examined the interventions from a healthcare perspective alone, a substantial part of the outcomes would have been ignored (HPSF: 49% of financial benefits, PAS: 59% of financial benefits). Jones et al. (2011) examined the SROI of the Food for Life programme, and also considered both health-related and non-health outcomes for local suppliers, school catering services, schools, parents, and local authorities (23). Financial proxies were defined by stakeholders, such as using the costs of a trip to the farm for valuing the knowledge of children about the origin of foods, which resulted in a SROI ratio of 4.4. Defining monetary values for non-financial outcomes is challenging as methods for obtaining proxies are not standardized. In the current study, we used standard cost prices only and refrained from defining financial proxies ourselves. This could have resulted in a conservative estimate of the SROI of HPSF and PAS. Due to the non-response on the parental questionnaire and the dynamic cohort design, our study suffered from missing data on covariates and longitudinal outcomes, which required multiple imputation.

Decisions on school-based lifestyle interventions should not be based on only the intervention's short-term return on investment. Follow-up assessments, as well as modeling to extrapolate short-term results beyond the trial period, are required to examine the full merits of school-based lifestyle interventions. Long-term information is, however, not always available for decision-making, because follow-up assessments are dependent on previous intervention implementation. To ensure successful intervention implementation and continuation it is crucial to have support from all stakeholders. The SROI framework allowed for comprehensively assessing the distribution of investments and outcomes over stakeholder groups. The results of the current study showed that the short-term benefits did not outweigh the investments of the HPSF initiative, but outcomes were generated for multiple sectors. The majority of investments were incurred by the education sector, while outcomes were received by the healthcare, household & leisure, and education sector. This information can be used as input for continuation decisions and investment strategies on the HPSF initiative by, for example, exploring alternative modes of intervention delivery (e.g., changing the organization of activities so that fewer pedagogical employees are needed), and examining whether a redistribution of investments over, amongst others, schools, parents, and the government is desired. The SROI framework can therefore serve as a tool in obtaining stakeholder support, foster intervention implementation and continuation, and facilitate follow-up research and decision-making on school-based lifestyle interventions. In accordance to others, we recommend that further research should focus on the valuation of outcomes in different sectors (18, 22, 24), and on the methods for valuing outcomes across different sectors (18) to further develop the methodology and enhance the implementation of the SROI methodology (17, 25). Future research is also needed to examine if SROI evaluations adequately meet the information needs of different stakeholders and optimally support the various decision-making processes on school-based lifestyle interventions.

## Data Availability Statement

The data that support the findings of this study were collected as part of the ‘Healthy Primary School of the Future’ quasi-experimental study. Data collection took place until 2019 to study the effects after 4 years of exposure. Data will become available from the authors upon reasonable request, following article publication on the 4-year effects and potential other comparative studies in the Netherlands.

## Ethics Statement

The need for ethical approval has been waived by the Medical Ethics Committee Zuyderland in Heerlen (MEC 14-N-142). All participants were required to complete an informed consent form in accordance with the Declaration of Helsinki, signed by both parents/caregivers, and by the children in case they are 12 years or older. The study protocol has been registered in the database ClinicalTrials.gov (NCT02800616).

## Author Contributions

MO and MJ designed the current study. MO, NB, and MW contributed to the data acquisition. MO and BW performed the statistical analyses. MO wrote the manuscript with input from all authors. All authors read and approved the final manuscript.

## Conflict of Interest

The authors declare that the research was conducted in the absence of any commercial or financial relationships that could be construed as a potential conflict of interest.

## Abbreviations

BMI: body mass index
HPSF: Healthy Primary Schools of the Future
HRQOL: health-related quality of life
PAS: Physical Activity Schools
PedsQl: Pediatric Quality of Life Inventory
QALY: quality-adjusted life year
SES: socioeconomic status
SROI: social return on investment
WTP: willingness to pay.

## References

[1] Lake A Townshend T. Obesogenic environments: exploring the built and food environments. J R Soc Promot Health. (2006) 126:262–7. 10.1177/1466424006070487 17152319

[2] Guthold R Stevens GA Riley LM Bull FC. Worldwide trends in insufficient physical activity from 2001 to 2016: a pooled analysis of 358 population-based surveys with 1.9 million participants. Lancet. (2018) 6:e1077–86. 10.1016/S2214-109X(18)30357-7 30193830

[3] Schönbeck Y Talma H van Dommelen P Bakker B Buitendijk SE Hirasing RA. Increase in prevalence of overweight in Dutch children and adolescents: a comparison of nationwide growth studies in 1980, 1997 and 2009. PLoS ONE. (2011) 6:e27608. 10.1371/journal.pone.0027608 22110687 PMC3216980

[4] Willeboordse M Jansen MW van den Heijkant SN Simons A Winkens B de Groot RH. The Healthy Primary School of the Future: study protocol of a quasi-experimental study. BMC Public Health. (2016) 16:639. 10.1186/s12889-016-3301-9 27456845 PMC4960894

[5] Bartelink NHM van Assema P Kremers SPJ Savelberg HH Oosterhoff M Willeboordse M. One- and two-year effects of the Healthy Primary School of the Future on children's dietary and physical activity behaviours: a quasi-experimental study. Nutrients. (2019) 11:689. 10.3390/nu11030689 30909515 PMC6470547

[6] Bartelink N Van Assema P Kremers SPJ Savelberg HH Oosterhoff M Willeboordse M. Can the Healthy Primary School of the Future offer perspective in the on-going obesity epidemic in young children?—A quasi-experimental study. BMJ Open. (2019) 9:e030676. 10.1136/bmjopen-2019-030676 31676651 PMC6830668

[7] Masters R Anwar E Collins B Cookson R Capewell S. Return on investment of public health interventions: a systematic review. J Epidemiol Community Health. (2017) 71:827–34. 10.1136/jech-2016-208141 28356325 PMC5537512

[8] Finkelstein EA Trogdon JG. Public health interventions for addressing childhood overweight: analysis of the business case. Am J Public Health. (2008) 98:411–5. 10.2105/AJPH.2007.114991 18235061 PMC2253570

[9] Laing CM Moules NJ. Social return on investment: a new approach to understanding and advocating for value in healthcare. J Nurs Adm. (2017) 47:623–8. 10.1097/NNA.0000000000000557 29135853

[10] Banke-Thomas AO Madaj B Charles A van den Broek N. Social return on investment (SROI) methodology to account for value for money of public health interventions: a systematic review. BMC Public Health. (2015) 15:582. 10.1186/s12889-015-1935-7 26099274 PMC4477315

[11] Bartelink NHM van Assema P Jansen MWJ Savelberg HH Willeboordse M Kremers SPJ. The Healthy Primary School of the Future: a contextual action-oriented research approach. Int J Environ Res Public Health. (2018) 15:2243. 10.3390/ijerph15102243 30720796 PMC6209969

[12] Nicholls J Lawlor E Neitzert E Goodspeed T A Guide to Social Return on Investment. (2012). Available online at: http://www.socialvalueuk.org (accessed March 1, 2019).

[13] Oosterhoff M Bosma H van Schayck OCP Joore MA. A cost analysis of school-based lifestyle interventions. Prev Sci. (2018) 19:716–27. 10.1007/s11121-018-0918-1 29856040 PMC6599187

[14] Bartelink NHM van Assema P Jansen MWJ Savelberg HH Moore GF Hawkins J. Process evaluation of the Healthy Primary School of the Future: the key learning points. BMC Public Health. (2019) 19:698. 10.1186/s12889-019-6947-2 31170941 PMC6554901

[15] Zorginstituut Nederland Kostenhandleiding: Methodologie van Kostenonderzoek en Referentieprijzen voor Economische Evaluaties in de Gezondheidszorg [Methodology of Cost Research and Cost Prices for Heal Economic Evaluations]. (2015). Available online at: https://www.zorginstituutnederland.nl/over-ons/werkwijzen-en-procedures/adviseren-over-en-verduidelijken-van-het-basispakket-aan-zorg/beoordeling-van-geneesmiddelen/richtlijnen-voor-economische-evaluatie (accessed November 14, 2019).

[16] Pomp M Schoemaker CG Polder JJ Op weg naar Maatschappelijke Kosten-Batenanalyses voor Preventie en zorg [Social Cost-Benefit Analysis for Prevention and Care]. Ministerie van Volksgezondheid (2014). Available online at: https://www.rivm.nl/publicaties/op-weg-naar-maatschappelijke-kosten-batenanalyses-voor-preventie-en-zorg-themarapport (accessed March 1, 2019).

[17] Drost R Paulus A Ruwaard D Evers S Handleiding Intersectorale Kosten en baten van (Preventieve) Interventies [Guideline for Intersectoral Costs and Benefits of Preventive Interventions]. (2014). Available online at: https://hsr.mumc.maastrichtuniversity.nl/sites/intranet.mumc.maastrichtuniversity.nl/files/hsr_mumc_maastrichtuniversity_nl/Symposia/30_Oct_2014_VGE_NVTAG/um-hsr_handleiding_intersectorale_kosten_en_baten.pdf (accessed November 25, 2019).

[18] Walker S Griffin S Asaria M Tsuchiya A Sculpher M. Striving for a societal perspective: a framework for economic evaluations when costs and effects fall on multiple sectors and decision makers. Appl Health Econ Health Policy. (2019) 17:577–90. 10.1007/s40258-019-00481-8 31098947 PMC6748888

[19] Oosterhoff M Bosma H van Schayck OCP Joore MA. Correction to: cost analysis of school-based lifestyle interventions. Prev Sci. (2019) 20:970–4. 10.1007/s11121-019-01030-4 31254132 PMC6828325

[20] Oosterhoff M Bosma H van Schayck OCP Evers S Dirksen CD Joore MA. A systematic review on economic evaluations of school-based lifestyle interventions targeting weight-related behaviours among 4–12 year olds: issues and ways forward. Prev Med. (2018) 114:115–22. 10.1016/j.ypmed.2018.06.015 29959951

[21] Goebbels AF Lakerveld J Ament AJ Bot SD Severens JL. Exploring non-health outcomes of health promotion: the perspective of participants in a lifestyle behaviour change intervention. Health Policy. (2012) 106:177–86. 10.1016/j.healthpol.2012.04.005 22575768

[22] van Mastrigt GA Paulus AT Aarts MJ Evers SM Alayli-Goebbels AF. A qualitative study on the views of experts regarding the incorporation of non-health outcomes into the economic evaluations of public health interventions. BMC Public Health. (2015) 15:954. 10.1186/s12889-015-2247-7 26399520 PMC4581076

[23] Jones RA Sinn N Campbell KJ Hesketh K Denney-Wilson E Morgan PJ. The importance of long-term follow-up in child and adolescent obesity prevention interventions. Int J Pediatr Obes. (2011) 6:178–81. 10.3109/17477166.2011.575155 21612335

[24] Sanders GD Neumann PJ Basu A Brock DW Feeny D Krahn M. Recommendations for conduct, methodological practices, and reporting of cost-effectiveness analyses: second panel on cost-effectiveness in health and medicine. JAMA. (2016) 316:1093–103. 10.1001/jama.2016.12195 27623463

[25] Hutchinson CL Berndt A Gilbert-Hunt S George S Ratcliffe J Valuing the impact of health and social care programmes using social return on investment analysis: how have academics advanced the methodology? A protocol for a systematic review of peer-reviewed literate. BMJ Open. (2018) 8:e022534 10.1136/bmjopen-2018-022534 PMC6303612 30530579

