# Supplementary material for: The Short-Term Value of the “Healthy Primary School of the Future” Initiative: A Social Return on Investment Analysis
Source: Front Public Health. 2020 Aug 21;8:401. doi: 10.3389/fpubh.2020.00401 (PMC7472552; doi:10.3389/fpubh.2020.00401)
Supplement: Supplementary file 1 [file Data_Sheet_1.docx]

Supplementary Material

# Appendix 1. Flowchart of children participating in the measurements

| Baseline  Total participating children: n=1403 (60.3% of all children) | | |
| --- | --- | --- |
| Selection for the current study  Participating children in classes 1-7: n=1255 | | |
| **HPSF (n=**361) | **PAS (n=**408) | **Control (n=**486) |
|  | | |
| One year follow up (Year 1)  Total participating children: n=1489 (60.7% of all children) | | |
| Selection for the current study  Participating children in classes 2-8: n=1455  Newly included (n=264); Drop-out* (n=64) | | |
| **HPSF (n=**469)  New included: n=132  Drop-out: n=24 | **PAS (n=**428)  New included: n=33  Drop-out: n=13 | **Control (n=**558)  New included: n=99  Drop-out: n=27 |
|  | | |
| Two year follow up (Year 2)  Total participating children: n=1470 (61.7% of all children) | | |
| Selection for the current study  Participating children in classes 3-8: n=1323  Newly included (n=158); Drop-out** (n=290) | | |
| **HPSF (n=**432)  New included: n=44  Drop-out: n=81 | **PAS (n=**376)  New included: n=38  Drop-out: n=90 | **Control (n=**515)  New included: n=76  Drop-out: n=119 |
|  | | |
| **Total participating children in study period: n=1974** | | |
| Selection for the current study**: n=**1676 | | |
| **HPSF (n=**537) | **PAS (n=**478) | **Control (n=**661) |

**Supplementary Figure 1.** The flow diagram is similar to Bartelink et al. (2019) (4). Notes: HPSF = Healthy Primary School of the Future; PAS = Physical Activity School; Year 1= after 12 months; Year 2 = after 24 months.* Reasons drop out Year 1: switched to other included school (n=2), other reasons e.g. moved out or actively stopped participation (n=62). ** Reasons drop out Year 2: finished school (n=228), switched to other included school (n=17), other reasons e.g. moved out or actively stopped participation (n=45).

# Appendix 2. Identifying and evidencing investments

In a previous study, we made an overview of the activities that were provided at HPSF and PAS in addition to the regular school curriculum (1). We calculated the corresponding net investments, which is the sum of all investments from the different sectors (see above) minus the cost offsets that were directly related to intervention delivery. Investments were either material costs (food, curriculum materials, monitoring equipment) or personnel costs (project and school coordinators, pedagogical staff, time costs of volunteers, compensation for external parties giving workshops). Direct cost offsets applied to HPSF, and included the offsets for food costs for the household sector as lunches were provided at school, and the value of the extended school day as the extended school hours at HPSF provided caregivers with additional time that could be spent on paid or unpaid work (productivity cost). In the first year of intervention implementation, there were also direct cost offsets for the household sector as the fee for the lunch break was not applied. Information on investments and cost offsets were retrieved from a literature review, stakeholder interviews, budget information, and curriculum information. Costs were measured in 2016 prices.

# Appendix 3. Identifying and evidencing outcomes

Table A3.1 details the outcome measures, the informants and sources, and the method of data collection. Outcomes that were measured qualitatively were obtained from semi-structured interview with stakeholders (see main text). Outcomes were measured quantitatively as part of the quasi-experimental study, with objective measurements on weight and height, child questionnaires, parental questionnaires, and school records (see main text) (2). To participate in data collection, participants completed an informed consent form signed by both parents/caregivers, and by the children in case they were 12 years or older (need for ethical approval has been waived by the Medical Ethics Committee Zuyderland in Heerlen).

A total of 1403 (T0), 1489 (T1), and 1470 (T2) children and their parents joined the study at the specific time points. Child questionnaires which were administered in grade 5-8, were completed by a total of 585 out of 1403 participants (41.7%) at T0 [not administered in eight grade at baseline], 843 out of 1489 participants (56.6%) at T1, and for 813 children out of 1470 (55.3%) at T2. The parental questionnaire was completed by 836 out of 1403 participants (59.6%) at T0, 725 out of 1489 (48.7%) at T1, and by 733 respondents out of 1470 (49.9%) at T2.

The study has a dynamic cohort design as children continually enter and leave primary school. Children and their parents were therefore also invited to join participate during the study. In the current study, we included a cohort of children that were enrolled at the participating schools and thus exposed to the interventions from baseline onwards. This included children from study year one to seven at baseline, children from study year two to eight at T1, and children from study year three to eight at T2. Children in grade 8 were excluded from the baseline measurement as no follow-up could be obtained.

We excluded the children that switched between schools between 2015 and 2017. For the current study, N=1676 children and their parents were included based on the selection of school years and school switchers excluded (see methods).

**Table A3.1** Outcomes and data collection

| **Sector** | **Outcome** | **Measurement*** | | **Valuation** | **Informants (questionnaire)** | | |
| --- | --- | --- | --- | --- | --- | --- | --- |
|  |  | **Quant.** | **Qual.** |  | **Child** | **Caregiver(s)** | **Other** |
| Healthcare | Health behaviors ^1^ | X |  | NA |  |  |  |
|  | Physical activity |  |  |  |  |  | Objective measurement |
|  | Leisure time physical activity |  |  |  | x | x |  |
|  | Food intake |  |  |  | x | x |  |
|  | Physical health | X |  |  |  |  |  |
|  | Height and weight |  |  |  |  |  | Objective measurement |
|  | HRQOL (EQ5D-Y questionnaire) | X |  | €36000 / QALY ^2^ | grade 5-8 | x |  |
|  | Medical resource use | X |  | see Table A1.3 ^4^ |  | x |  |
|  | Parental and teacher practices towards nutrition and PA | X | X | NA |  | x | Interviews |
|  | HRQOL family (EQ5D questionnaire) | X |  | €36000 / QALY ^2^ |  | x |  |
| Education | School behaviors |  | X | NA |  |  | Interviews |
|  | Cognitive performances |  |  |  |  |  |  |
|  | Test scores | X |  | NA |  |  | School data |
|  | Absenteeism from school | X |  | €26.48 / day ^3^ |  |  | School records |
| Household & leisure | Household expenses on nutrition | NA | NA |  |  |  |  |
|  | Sports club membership | X |  | NA | x | x |  |
|  | Engagement with the local community |  | X | NA |  |  | Interviews |
| Labour & social security | Opportunities for caregivers to engage in work/activities | X |  | Paid work: €35.53 / hour  Unpaid work: €14.31 / hour ^4^ |  | x |  |
|  | Opportunities for local employment | NA | NA |  |  |  |  |
|  | Parental leave and absenteeism from work due to sickness of the child | X |  | Paid work: €35.53 / hour  Unpaid work: €14.31 / hour ^4^ |  | x |  |

**Table A3.1** [continued]

| **Sector** | **Outcome** | **Measurement*** | | **Valuation** | | **Informants (questionnaire)** | | |
| --- | --- | --- | --- | --- | --- | --- | --- | --- |
|  |  | **Quant.** | **Qual.** |  | | **Child** | **Caregiver(s)** | **Other** |
| Intangibles | Non-cognitive skills |  |  |  |  | |  |  |
|  | Self-efficacy (SEQ-C, Maniken scale) | X |  | NA | grade 5-8 | |  |  |
|  | Social skills |  | X | NA |  | |  | Interviews |
|  | Psychosocial health |  |  |  |  | |  |  |
|  | Quality of life (PedsQL instrument) ^5^ | X |  | NA | grade 5-8 | | x |  |
|  | Psychosocial functioning (SDQ) ^6^ | X |  | NA |  | | x |  |
|  | Parental wellbeing (SWLS) | X |  | NA |  | | x |  |
|  | Satisfaction with school | X |  | NA |  | |  | School surveys (standardized measurement) |

**Supplementary Table A3.1** Notes: EQ5D-Y = EuroQol- 5 Dimensions Youth questionnaire; HRQOL = health-related quality of life; PedsQL = Pediatric Inventory of Quality of Life; SEQ-C = self-efficacy questionnaire for children; SDQ = Strengths and Difficulties Questionnaire; SWLS = Satisfaction with Life Scale; qual. = qualitative; quant.= quantitative.
^1^ Papers of Bartelink et al. (2019) (3, 4).
^2^ Pomp, M., Schoemaker, C.G., Polder, J.J. (2014). Op weg naar maatschappelijke kosten-batenanalyses voor preventie en zorg [Social cost-benefit analysis for prevention and care]. Ministerie van Volksgezondheid. Available online at: https://www.rivm.nl/publicaties/op-weg-naar-maatschappelijke-kosten-batenanalyses-voor-preventie-en-zorg-themarapport. [Accessed March 1, 2019]. (5).

^3^ Drost, R., Paulus, A., Ruwaard, D., Evers, S. (2014). Handleiding intersectorale kosten en baten van (preventieve) interventies [Guideline for intersectoral costs and benefits of preventive interventions]. Available online at: https://hsr.mumc.maastrichtuniversity.nl/sites/intranet.mumc.maastrichtuniversity.nl/files/hsr_mumc_maastrichtuniversity_nl/Symposia/30_Oct_2014_VGE_NVTAG/um-hsr_handleiding_intersectorale_kosten_en_baten.pdf. [Accessed November 25, 2019]. (6).

^4^ Dutch guidelines for costing in health economic evaluations (7).

^5^ Including physical, emotional, social, and school functioning.

^6^ Including emotional, hyperactivity/inattention, conduct, and peer relationship difficulties.

* Measurement procedures did not differ between the intervention groups (HPSF and PAS) and control schools.

# Appendix 4. SROI assessment

**Outcomes expressed in the SROI calculation and SROI story**The reasons for excluding outcomes from the SROI calculation are further detailed in Table A4.1. The details on the outcomes for the SROI calculation are provided in Table A4.2.

**Table A4.1** Details on outcomes included in the SROI story

| **Sector** | **See Fig 1** | **Outcomes** | **Justification for exclusion from SROI calculation** |
| --- | --- | --- | --- |
| Healthcare | H1 | Health behaviors (3)   - Physical activity - Leisure time physical activity - Food intake | When including all impacts for the healthcare sector, this would result in double counting as it is assumed that changes in children’s health behaviours and health status affect their HRQOL and medical resource use. As such, changes in health behaviours and physical health are intermediate outcomes for the impacts on HRQOL and medical resource use. |
|  | H2 | Physical health (4)   - Height and weight |  |
|  | H5 | Parental and teacher practices towards nutrition and PA | Including the impacts on health behaviours and HRQOL could lead to double counting of outcomes. It is assumed that the changes in practices towards nutrition and PA are an intermediate outcome of the potential impact on family HRQOL. |
| Education | E1 | School behaviours (e.g. bullying) | Measured qualitatively. |
|  | E2 | Executive functioning and cognitive performance | A longer time horizon is required to examine the potential financial returns. |
|  | E4,E5 | School satisfaction | No financial proxy available to express the effects in financial returns. |
| Intangibles | I1 | Non-cognitive skills |  |
|  |  | - Self-efficacy | No financial proxy available to express the effects in financial returns. |
|  |  | - Social skills | Measured qualitatively. |
|  | I2 | Psychosocial health and wellbeing |  |
|  |  | - Quality of life (PedsQL instrument) | No financial proxy available to express the effects in financial returns.  No financial proxy available to express the effects in financial returns.  No financial proxy available to express the effects in financial returns. |
|  |  | - Psychosocial functioning (SDQ) |  |
|  | I3 | Wellbeing family (SWLS) |  |
| Household & leisure | HL1 | Household expenses on healthy nutrition | Not formally measured within the quasi-experimental study. |
|  | HL2 | Sports club membership | No financial proxy available to express the effects in financial returns. |
|  | HL3 | Engagement with the local community | Measured qualitatively. |
| Labour & social security | L1 | Opportunities for local employment | Not formally measured within the quasi-experimental study. |

**Supplementary Table A4.1** Notes: HRQOL = health-related quality of life; PA = physical activity; PedsQL = Pediatric Inventory of Quality of Life; SEQ-C = self-efficacy questionnaire for children; SDQ = Strengths and Difficulties Questionnaire; SWLS = Satisfaction with Life Scale.

**Table A4.2** Details on outcomes included in the SROI calculation

| **Sector** | **See Fig 1** | **Outcomes** | **Measurement** | **Valuation** |
| --- | --- | --- | --- | --- |
| Healthcare | H3 | HRQOL | Measured with the EQ5D-Y questionnaire, self-report for children in grade 5-8, proxy report by caregivers for children <grade 5. | €36000 / QALY ^1^ |
|  | H4 | Medical resource use | Included in the parental questionnaire ^2^ | see Table A1.3 ^3^ |
|  | H6 | HRQOL family | Measured with the EQ5D questionnaire | €36000 / QALY ^1^ |
| Education | E3 | Absenteeism from school | School records | €26.48 / day ^4^ |
| Household & leisure | HL4 | Opportunities for caregivers to engage in work/activities ^5^ | The volume was based on the duration of the extended school day | Paid work: €35.53 / hour  Unpaid work: €14.31 / hour ^3^ |
| Labour & social security | L2 | Parental leave and absenteeism from work due to sickness of the child | *Parental questionnaire ^6^* | *NA ^6^* |

**Supplementary Table A4.2** Notes: EQ5D-Y = EuroQol- 5 Dimensions Youth questionnaire; HRQOL = health-related quality of life.
^1^ Pomp, M., Schoemaker, C.G., Polder, J.J. (2014). Op weg naar maatschappelijke kosten-batenanalyses voor preventie en zorg [Social cost-benefit analysis for prevention and care]. Ministerie van Volksgezondheid. Available online at: https://www.rivm.nl/publicaties/op-weg-naar-maatschappelijke-kosten-batenanalyses-voor-preventie-en-zorg-themarapport. [Accessed March 1, 2019]. (5).

^2^ Healthcare resource use was measured by a parental questionnaire with a 12-month recall about the number of healthcare visits (including visits to the general practice and physicians, and use of mental health and youth care services), hospital admissions and medication use.

^3^ Dutch guidelines for costing in health economic evaluations (7).

^4^ Drost, R., Paulus, A., Ruwaard, D., Evers, S. (2014). Handleiding intersectorale kosten en baten van (preventieve) interventies [Guideline for intersectoral costs and benefits of preventive interventions]. Available online at: https://hsr.mumc.maastrichtuniversity.nl/sites/intranet.mumc.maastrichtuniversity.nl/files/hsr_mumc_maastrichtuniversity_nl/Symposia/30_Oct_2014_VGE_NVTAG/um-hsr_handleiding_intersectorale_kosten_en_baten.pdf. [Accessed November 25, 2019]. (6).

^5^ In the calculation of the investments of HPSF, the consequences of the extended school day were included in terms of increased parental productivity. The opportunities for caregivers to engage in work (parental productivity) could not only be affected by the extended school hours at HPSF, but could also be affected by child and parental health and HRQOL.

^6^ Due to the small numbers (<10%), the potential benefits could not be reliably estimated and we refrained from including them in the calculation of financial return on investment.

## Calculation of benefits for the social return on investment calculation

Benefits are the product of outcomes (volume) and the financial proxy or unit cost. The reported volumes after year 1 and year 2 were combined to calculate the aggregated results over the total follow-up period of two years. The aggregated volumes were then multiplied with the unit costs (2017 prices).

There were two methods for summing up volumes:

1) When volumes were obtained with a recall period of 12 months, volumes were summed up:

$$V_{i}=v_{i,y1}+v_{i,y2}$$

where V represents the total volume, y1 is the 12 month period from baseline to T1, and y2 is the 12 month period from T1 to T2.

2) When volumes were obtained for the specific time points, volumes were summed up with the area under the curve method:

$$V_{i}=\left( \frac{v_{i, t0}+v_{i,t1}}{2} \right)*\left( t1-t0 \right)+\left( \frac{v_{i,t1}+ v_{i,t2}}{2} \right)*\left( t2-t1 \right)$$

where V represents the total volume, t0 the time point of the baseline measurement, t1 the time point of the first follow-up measurement (after 12 months), and t2 the time point of the first follow-up measurement (after 24 months).

### H3: HR-Qol of the child

Children’s HR-QoL was measured by the EuroQol 5 dimensions Youth questionnaire (EQ-5D-Y) which was completed by children in grade 5-8 (8-12 year-olds), and by the EQ5-5D-Y proxy questionnaire which was completed by parental report. In the base-case analysis, we used the self-reports on HR-QoL in 8-12 year-olds and proxy-reports for children between 4-8 years of age. The EQ5D questionnaire contains five questions regarding mobility, self-care, usual activities, pain/discomfort, and anxiety/depression, scored on a 3-point (child and proxy questionnaire) or 5-point (carer) rating scale. The corresponding health states were converted to utility values with valuations obtained from the general public (8). Quality-adjusted life years (QALYs) were calculated by means of the area under the curve method, in which the time in a certain health state was multiplied by the utility value (see method 2). With this method it is assumed that the utility between two consecutive measurements equals the mean of those two measurements. QALYs were valued using a financial proxy of €36000 per QALY (5).

### H4: Medical resource use

Healthcare resource use was measured by a parental questionnaire with a 12-month recall about the number of healthcare visits (including visits to the general practice and physicians, and use of mental health and youth care services), hospital admissions and medication use (see method 1). The costs of medication use were calculated according to the recommendations of the Dutch guidelines for costing in health economic evaluations (7). Information on the costs of pharmaceuticals was obtained from the website of the Dutch healthcare institute (www.medicijnkosten.nl).

| **Resources** | **Unit costs^3^** |
| --- | --- |
| GP visits | €35.53 ^B^ |
| Speech therapist visits | €30.49 ^B^ |
| Specialist visits | €92.47 ^B^ |
| Physiotherapist / Occupational therapist visits | €33.53 ^B^ |
| Youth care visits (Dutch: jeugdzorg en jeugdhulpverlening) | €62.24 ^C^ |
| Psychologist / social worker visits | €65.04 ^B^ |
| Hospital admissions days | €483.71 ^B^ |
| Costs of prescribed medication | See methods |

**Table A4.3 Unit costs for medical resource use.**

**Supplementary Table A4.3** ^A^ Guideline for intersectoral costs and benefits of preventive interventions (OCW kerncijfers 2007-2011) (6). ^B^ Dutch guidelines for costing in health economic evaluations (7). ^C^ [Inzicht in tarieven WMO en jeugdzorg Fase 2. KPMG] (9).

### H6: HRQOL family

HR-QoL of carers (one respondent per child) was assessed with the EuroQol 5 dimensions 5 levels questionnaire (EQ-5D-5L). Health states were converted to utility values with valuations obtained from the general public. Quality-adjusted life years (QALYs) were calculated by means of the area under the curve method (see method 2). QALYs were valued using a financial proxy of €36000 per QALY (5).

### E3: Absenteeism from school

School absenteeism days, with a recall period of one school year, were retrieved from school records (see method 1). Annual school absenteeism days were calculated, with making a distinction between health-related school absenteeism and non-health related school absenteeism. School absenteeism days were valued with the standard cost price (6).

### L2: Parental leave and absenteeism from work

Parental leave and absenteeism from work or education were measured as the annual leave or absenteeism days from work or education due to the illness of the child. Leave and absenteeism days were measured with a recall period of 12 months, for both the primary caregiver and the partner (see method 1). Productivity losses associated with absenteeism from work or education were valued using the friction cost method using an average of 8 working hours per day (7).

### HL4: Opportunities for caregivers to engage in work/activities

Parental labour participation was measured as the number of annual working hours for paid work and unpaid work for the primary caregiver and the partner, for each time point specifically (see method 2). The costs of unpaid labour were valued with the proxy good method which values unpaid labour at the costs of housekeeping services, representing a close market substitute of unpaid labour (7). We used an average of 45 working weeks per year to calculate the value of parental productivity.

## Statistical analysis

Multiple imputation was used to account for possible selective non-response (missing at random assumption) and to use all available data. Imputations were generated with the MICE package in R using 50 imputed datasets with 20 iterations. Missing data on covariates were predicted by all other variables measured at baseline. Missing data on outcome variables were imputed for each time point using all covariates and the specific outcome variable at other time points. Total healthcare costs were defined as the sum of all cost categories, where imputation was performed at the level of cost categories (see Table 1 – Medical Resource Use).

The mean differences in aggregated benefits over year 1 and year 2 were examined with a generalized linear model with a Gamma distribution and a log link function to account for the zero values and skewness of the data. The analyses were adjusted for sex, study year at baseline, socioeconomic status (SES), ethnicity, baseline BMI z-scores, and baseline outcome scores. Children’s SES was calculated as the mean of standardized scores on maternal education level, paternal educational level, and household income (adjusted for household size) which were obtained from the parental questionnaire (10). The mean scores were categorized in low, middle and high SES scores based on tertiles. Children’s ethnicity (native background, Western background, non-Western background) was determined by the country of birth of both parents (11). BMI z-scores were calculated by using Dutch reference values (12).

## Scenario and sensitivity analysis

Scenario analyses were performed to analyze the SROI of HPSF and PAS for specific situations: 1) First of all, we assumed that 8 instead of 12 pedagogical workers would be needed at HPSF when children from different grades would not have lunch at the same time and when pedagogical staff, teachers, and teaching assistants would share/shift tasks. 2) In a second scenario, we included lower investments for HPSF and PAS for e.g. coordination and the lunch that are expected occur in a so-called steady state due to efficiency improvements and learning effects (1, 13). 3) Thirdly, we excluded children who are in grade 7 at baseline and thus are leaving school during the 2-year study period. This analysis was used to examine whether the SROI would be different when HPSF and PAS were only offered to children who received the interventions for multiple years during the school-age period, compared to children who were nearly leaving school.

Sensitivity analyses were conducted to see how the results would change under different assumptions: 1) Including spillovers on parental HRQOL and productivity. Spillover effects occur when a persons’ actions or behaviors indirectly affect other persons’ outcomes through for example peer effects or social interactions. It was assumed that HPSF and PAS would first impact on outcomes in the child, which may subsequently transfer to impacts in the household. Previous studies found that school health promotion directed at children was effective in improving lifestyle behaviors and health outcomes of family members (14, 15). In the calculation of the investments of HPSF, the consequences of the extended school day were already included in terms of increased parental productivity. It is, however, likely that parental productivity may also be affected by children’s and caregivers’ HRQOL (Figure 1: L2 and HL4). Due to the small numbers (<10 percent) for parental absenteeism from work (Figure 1: L2), the potential benefits could not be reliably estimated and we refrained from including them in the calculation of financial return on investment. 2) No offsets in the investment costs of HPSF due to the extended school day. In the base case analysis we included the effects of the extended school day at HPSF on the opportunities for caregivers to engage in working activities. In the sensitivity analysis we excluded these offsets due to the uncertainty of this outcome (see costing study (1, 13). 3 & 4) Willingness to pay (WTP) thresholds of €20.000 and €50.000 per QALY gained instead of €36.000 per QALY gained. No formal thresholds exist for the willingness to pay per QALY gain in the area of prevention. Although it is advised to use a WTP threshold of €36.000 per QALY gained for social cost-benefit analyses in the Netherlands (5), an alternative threshold of €20.000 per QALY gained has been mentioned (16). We analyzed the SROI for a WTP threshold of €20.000 and €50.000 per QALY gained. 6) No discounting of investments and benefits. 5) A complete case analysis (non-imputed outcomes).

| **Scenario and sensitivity analyses** | | **SROI outcome** | **HPSF vs. control schools** | | **PAS vs. control schools** | |
| --- | --- | --- | --- | --- | --- | --- |
|  |  |  | Estimate | (95% CI) | Estimate | (95% CI) |
|  | Base case analysis | Net investment | 859 |  | 1017 |  |
|  |  | Benefits | 8 | (-1085;1057) | 49 | (-1041;1097) |
|  |  | Net monetary benefit | -851 | (-1945;198) | -968 | (-2058;80) |
|  |  | Ratio of benefits to investment | 0.01 | (-1.3;1.2) | 0.05 | (-1.0;1.1) |
|  |  | Net monetary benefit / child / day ^1^ | -2.66 | (-6.08;0.62) | -3.03 | (-6.43;0.25) |
| **Scenario analyses** | 8 instead of 12 pedagogical employees at HPSF | Net investment | 497 |  | 1017 |  |
|  |  | Benefits | 8 | (-1085;1057) | 49 | (-1041;1097) |
|  |  | Net monetary benefit | -488 | (-1582;560) | -968 | (-2058;80) |
|  |  | Ratio of benefits to investment | 0.02 | (-2.2;2.1) | 0.10 | (-1.0;1.1) |
|  |  | Net monetary benefit / child / day ^1^ | -1.53 | (-4.94;1.75) | -3.03 | (-6.43;0.25) |
|  | Lower investments for HPSF and PAS expected to occur on the longer-term (steady state) | Net investment | 318 |  | 719 |  |
|  |  | Benefits | 8 | (-1085;1057) | 49 | (-1041;1097) |
|  |  | Net monetary benefit | -309 | (-1403;740) | -670 | (-1760;378) |
|  |  | Ratio of benefits to investment | 0.03 | (-3.4;3.3) | 0.15 | (-1.4;1.5) |
|  |  | Net monetary benefit / child / day ^1^ | -0.97 | (-4.38;2.31) | -2.09 | (-5.50;1.18) |
|  | Excluding children in grade 7 at baseline, who are nearly leaving school | Net investment | 859 |  | 1017 |  |
|  |  | Benefits | 1482 | (-1418;3929) | 708 | (-2134;3293) |
|  |  | Net monetary benefit | 622 | (-2277;3070) | -309 | (-3151;2276) |
|  |  | Ratio of benefits to investment | 1.70 | (-1.6;4.6) | 0.70 | (-2.1;3.2) |
|  |  | Net monetary benefit / child / day ^1^ | 1.94 | (-7.12;9.59) | -0.97 | (-9.85;7.11) |
| **Sensitivity analyses** | Including spillovers on caregiver’s HRQOL and productivity (Appendix 2: unit costs) | Net investment | 859 |  | 1017 |  |
|  |  | Benefits | 40 | (-2969;3631) | 739 | (-2894;3733) |
|  |  | Net monetary benefit | -820 | (-3829;2772) | -278 | (-3911;2716) |
|  |  | Ratio of benefits to investment | 0.05 | (-3.5;4.2) | 0.70 | (-2.8;3.7) |
|  |  | Net monetary benefit / child / day ^1^ | -2.56 | (-11.96;8.66) | -0.87 | (-12.22;8.49) |

# Appendix 5. Scenario and sensitivity analysis

**Table A5.1** Scenario and sensitivity analyses (€/child/ 2 years).

**Table A5.1 [continued]**

| **Scenario and sensitivity analyses** | | **SROI outcome** | **HPSF vs. control schools** | | **PAS vs. control schools** | |
| --- | --- | --- | --- | --- | --- | --- |
|  |  |  | Estimate | (95% CI) | Estimate | (95% CI) |
| **Sensitivity analyses** | No delivery-related offsets in social opportunity costs of HPSF due to the extended school day | Net investment | 2025 |  | 1017 |  |
|  |  | Benefits | 8 | (-1085;1057) | 49 | (-1041;1097) |
|  |  | Net monetary benefit | -2017 | (-3111;-968) | -968 | (-2058;80) |
|  |  | Ratio of benefits to investment | 0.00 | (-0.5;0.5) | 0.05 | (-1.0;1.1) |
|  |  | Net monetary benefit / child / day ^1^ | -6.30 | (-9.72;-3.02) | -3.03 | (-6.43;0.25) |
|  | Willingness to pay threshold of €20.000/QALY gained instead of €36.000/QALY gained | Net investment | 859 |  | 1017 |  |
|  |  | Benefits | 8 | (-506;478) | 49 | (-462;517) |
|  |  | Net monetary benefit | -851 | (-1365;-382) | -968 | (-1479;-500) |
|  |  | Ratio of benefits to investment | 0.01 | (-0.6;0.6) | 0.05 | (-0.5;0.5) |
|  |  | Net monetary benefit / child / day ^1^ | -2.66 | (-4.27;-1.19) | -3.03 | (-4.62;-1.56) |
|  | Willingness to pay threshold of €50.000/QALY gained  instead of €36.000/QALY gained | Net investment | 859 |  | 1017 |  |
|  |  | Benefits | 8 | (-1593;1565) | 49 | (-1548;1604) |
|  |  | Net monetary benefit | -851 | (-2452;705) | -968 | (-2565;587) |
|  |  | Ratio of benefits to investment | 0.01 | (-1.9;1.8) | 0.05 | (-1.5;1.6) |
|  |  | Net monetary benefit / child / day ^1^ | -2.66 | (-7.66;2.20) | -3.03 | (-8.02;1.83) |
|  | No discounting instead of an annual discount rate of  2.5% for costs and outcomes | Net investment | 828 |  | 980 |  |
|  |  | Benefits | 9 | (-1140;1111) | 51 | (-1094;1152) |
|  |  | Net monetary benefit | -820 | (-1969;282) | -929 | (-2074;172) |
|  |  | Ratio of benefits to investment | 0.01 | (-1.4;1.3) | 0.05 | (-1.1;1.2) |
|  |  | Net monetary benefit / child / day ^1^ | -2.56 | (-6.15;0.88) | -2.90 | (-6.48;0.54) |
|  | Complete case analysis | Net investment | 859 |  | 1017 |  |
|  |  | Benefits | -1581 | (-3588;264) | 582 | (-1527;2564) |
|  |  | Net monetary benefit | -2440 | (-4447;-596) | -435 | (-2544;1547) |
|  |  | Ratio of benefits to investment | -1.80 | (-4.2;0.3) | 0.60 | (-1.5;2.5) |
|  |  | Net monetary benefit / child / day ^1^ | -7.62 | (-13.90;-1.86) | -1.36 | (-7.95;4.84) |

**Supplementary Table A5.1** ^1^ For a total of 160 schooldays per year (total of 320 days for two years).
Notes: CI = confidence interval; HPSF **=** Healthy Primary School of the Future; INB = incremental net benefit; PAS = Physical Activity School; SROI = social return on investment; WTP = willingness to pay; QALY = quality-adjusted life year.

| **Panel A: Net investments** | | | | | | | | | | | | **HPSF versus control schools** | | | | | | | |
| --- | --- | --- | --- | --- | --- | --- | --- | --- | --- | --- | --- | --- | --- | --- | --- | --- | --- | --- | --- |
|  |  |  |  |  |  |  |  |  |  |  |  | € per child (discounted results) | | | | | | | |
| Net investment year 1 |  |  |  |  | |  | |  | | |  | 440 |  | |  | | | |  |
| Net investment year 2 |  |  |  |  | |  | |  | | |  | 420 |  | |  | | | |  |
| **Total net investments (year 1 and year 2) ^1^** | | | | | | | | | | |  | **859** |  | |  | | | |  |
| **Panel B: Financial returns** | **Unit cost** | **Control schools** | | | **HPSF** | |  | | **PAS** | |  | **HPSF versus control schools** | | | | | | | |
|  |  |  | | |  | |  | |  | |  | **Rate ratio ^2^** | | | | **Benefits €** per child Y1+Y2 **^3^** | | | |
|  |  | Mean | (SE) | | Mean | | (SE) | | Mean | | (SE) | Estimate | | (95% CI) | | Estimate | | (95% CI) | |
| QALYs child | €36000 /QALY ^4^ | 68508 | (335.0) | | 68554 | | (332.6) | | 68531 | | (319.6) | 1.00 | | (0.98;1.02) | | 0 | | (-1304;1304) | |
| Medical resource use | See Appendix 1^5^ | -1056 | (211.3) | | -997 | | (204.9) | | -1010 | | (194.4) | 0.98 | | (0.81;1.18) | | 20 | | (-180;191) | |
| QALYs primary caregiver | €36000 /QALY ^4^ | 64339 | (501.9) | | 64567 | | (519.8) | | 64604 | | (531.1) | 1.00 | | (0.97;1.04) | | 0 | | (-1837;2449) | |
| HR absenteeism | €26.48 /day ^5,6^ | -338 | (15.6) | | -352 | | (17.6) | | -309 | | (15.9) | 1.05 | | (0.92;1.19) | | -16 | | (-61;26) | |
| Other school absenteeism | €26.48 /day ^5,6^ | -31 | (6.8) | | -25 | | (7.4) | | -29 | | (6.6) | 0.85 | | (0.93;1.16) | | 4 | | (-5;2) | |
| Parental time spent at paid work/week | €35.53 /hour ^7^ | 1639 | (42.1) | | 1705 | | (41.4) | | 1786 | | (43.1) | 1.02 | | (0.97;1.08) | | 31 | | (-47;125) | |
| **Total benefits (total year 1 and year 2)** | | | | | | | | | | |  |  | |  | | **40** | | **(-2969;3631)** | |
| **Panel C: Social return on investment** | | | | | | | | | | | | **HPSF versus control schools** | | | | | | | |
|  |  |  |  |  |  |  |  |  |  |  |  |  | |  | | | **€** per child Y1+Y2 | | |
|  |  |  |  |  |  |  |  |  |  |  |  |  | |  | | | Estimate | | (95% CI) |
| Ratio of benefits to investment ^8^ | | | | | | | | | |  | |  | |  | | | 0.05 | | (-3.5;4.2) |
| Net monetary benefit ^9^ | | | | | | | | | |  | |  | |  | | | -820 | | (-3829;2772) |
| Net monetary benefit per child per day ^10^ | | |  |  | |  | |  | |  | |  | |  | | | -2.56 | | (-11.96;8.66) |

**Table A5.2** Total benefits (€) including spillovers in year 1 and year 2 (N=1676, adjusted for covariates).

| **Panel A: Net investments** | | | | | | | | | | | **PAS versus control schools** | | | |
| --- | --- | --- | --- | --- | --- | --- | --- | --- | --- | --- | --- | --- | --- | --- |
|  |  |  |  |  |  |  |  |  |  |  | € per child (discounted results) | | | |
| Net investment year 1 |  |  |  |  | |  | |  | |  | 518 |  |  |  |
| Net investment year 2 |  |  |  |  | |  | |  | |  | 499 |  |  |  |
| **Total net investments (year 1 and year 2) ^1^** | | | | | | | | | |  | **1,017** |  |  |  |
| **Panel B: Financial returns** | **Unit cost** | **Control schools** | | | **HPSF** |  | | **PAS** | |  | **PAS versus control schools** | | | |
|  |  |  | | |  |  | |  | |  | **Rate ratio ^2^** | | **Benefits €** per child Y1+Y2 **^3^** | |
|  |  | Mean | (SE) | | Mean | (SE) | | Mean | | (SE) | Estimate | (95% CI) | Estimate | (95% CI) |
| QALYs child | €36000 /QALY ^4^ | 68508 | (335.0) | | 68554 | (332.6) | | 68531 | | (319.6) | 1.00 | (0.98;1.02) | 0 | (-1304;1304) |
| Medical resource use | See Appendix 1^5^ | -1056 | (211.3) | | -997 | (204.9) | | -1010 | | (194.4) | 0.98 | (0.81;1.18) | 20 | (-180;191) |
| QALYs primary caregiver | €36000 /QALY ^4^ | 64339 | (501.9) | | 64567 | (519.8) | | 64604 | | (531.1) | 1.01 | (0.97;1.04) | 612 | (-1837;2449) |
| HR absenteeism | €26.48 /day ^5,6^ | -338 | (15.6) | | -352 | (17.6) | | -309 | | (15.9) | 0.92 | (0.81;1.06) | 26 | (-19;61) |
| Other school absenteeism | €26.48 /day ^5,6^ | -31 | (6.8) | | -25 | (7.4) | | -29 | | (6.6) | 0.90 | (0.63;1.24) | 3 | (-7;11) |
| Parental time spent at paid work/week | €35.53 /hour ^7^ | 1639 | (42.1) | | 1705 | (41.4) | | 1786 | | (43.1) | 1.05 | (0.99;1.12) | 78 | (-16;187) |
| **Total benefits (total year 1 and year 2)** | | | | | | | | | |  |  |  | **739** | **(-2894;3733)** |
| **Panel C: Social return on investment** | | | | | | | | | | | **PAS versus control schools** | | | |
|  |  |  |  |  |  |  |  |  |  |  |  |  | **€** per child Y1+Y2 | |
|  |  |  |  |  |  |  |  |  |  |  |  |  | Estimate | (95% CI) |
| Ratio of benefits to investment ^8^ | | | | | | | | |  | |  |  | 0.7 | (-2.8;3.7) |
| Net monetary benefit ^9^ | | | | | | | | |  | |  |  | -278 | (-3911;2716) |
| Net monetary benefit per child per day ^10^ | | |  |  | |  |  | |  | |  |  | -0.87 | (-12.22;8.49) |

**Table A5.2** [continued]

**Supplementary Table A5.2** ^1^ Net investment = investments minus delivery-related offsets (HPSF: household expenses on lunches for children, and the value of the extended school day for parental productivity). ^2^ Ratio of mean benefits for HPSF or PAS versus control schools.
^3^ Benefits for HPSF or PAS = mean value at control schools * rate ratio (repeated for lower and upper bound of the confidence interval). Discounted with an annual discount rate of

2.5% to account for differential timing of investments and financial returns.
^4^ Pomp, M., Schoemaker, C.G., Polder, J.J. (2014). Op weg naar maatschappelijke kosten-batenanalyses voor preventie en zorg [Social cost-benefit analysis for prevention and care]. Ministerie van Volksgezondheid. Available online at: https://www.rivm.nl/publicaties/op-weg-naar-maatschappelijke-kosten-batenanalyses-voor-preventie-en-zorg-themarapport. [Accessed March 1, 2019]. (5).
^5^ Because medical resource use and school absenteeism represent a cost, they are represented as a negative financial return. ^6^ Drost, R., Paulus, A., Ruwaard, D., Evers, S. (2014). Handleiding intersectorale kosten en baten van (preventieve) interventies [Guideline for intersectoral costs and benefits of preventive interventions]. Available online at: https://hsr.mumc.maastrichtuniversity.nl/sites/intranet.mumc.maastrichtuniversity.nl/files/hsr_mumc_maastrichtuniversity_nl/Symposia/30_Oct_2014_VGE_NVTAG/um-hsr_handleiding_intersectorale_kosten_en_baten.pdf. [Accessed November 25, 2019]. (6). ^7^ Dutch guidelines for costing in health economic evaluations (7). ^8^ Ratio of total of benefits and investments. ^9^ Incremental net monetary benefit = incremental benefits – incremental net investments.
^10^ For a total of 160 schooldays per year (total of 320 days for two years).
Notes: CI = confidence interval; HPSF = Healthy Primary School of the Future; HR = health-related; IQR = interquartile range; PAS = Physical Activity School; QALYs = quality-adjusted life years; SE= standard error.

**Figure A5.1** Point estimates of the investments and benefits for the scenario and sensitivity analyses (HPSF).


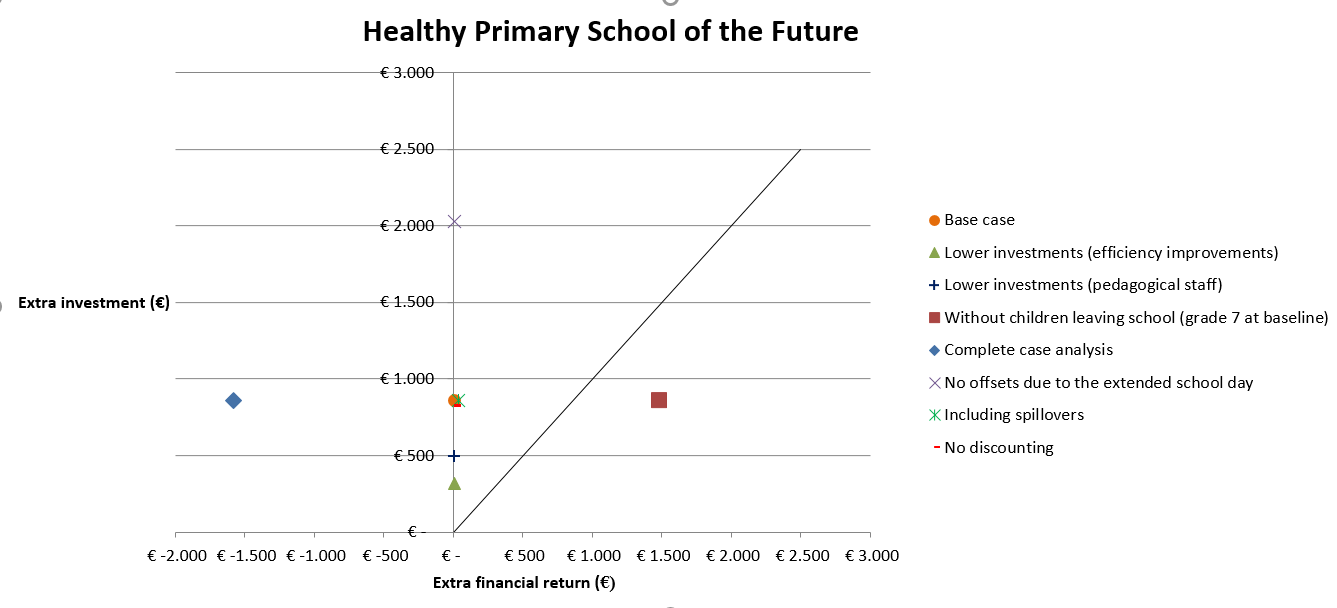


**Supplementary Figure A5.1** Notes: HRQOL = health-related quality of life; QALY = quality-adjusted life year**;** WTP = willingness to pay.
The line represents a SROI ratio of 1. For all points above the line the ratio of benefits / investments <1. For all points under the line the ratio of benefits / investments > 1.


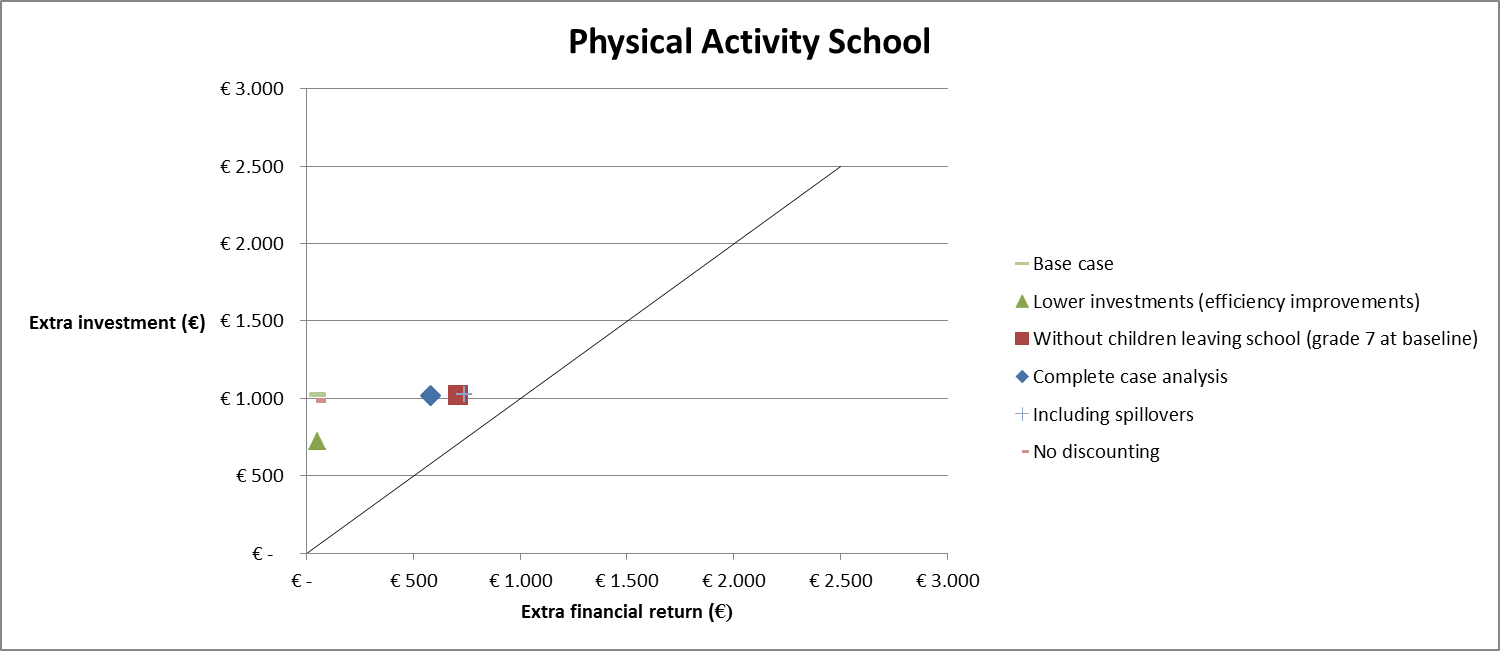
**Figure A5.2** Point estimates of the investments and benefits for the scenario and sensitivity analyses (PAS).
 **Supplementary Figure A5.2** Notes: HRQOL = health-related quality of life; QALY = quality-adjusted life year; WTP = willingness to pay.
The line represents a SROI ratio of 1. For all points above the line the ratio of benefits / investments <1. For all points under the line the ratio of benefits / investments > 1.**Figure A5.3** Point estimates and uncertainty of the around the benefits for the scenario and sensitivity analyses (HPSF).


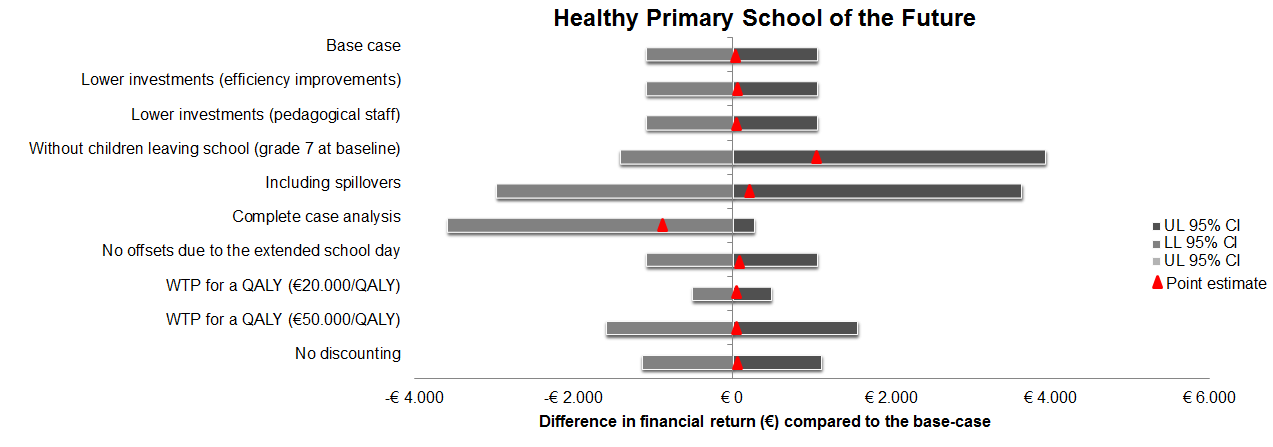


**Supplementary Figure A5.3** Notes: HRQOL = health-related quality of life; QALY = quality-adjusted life year; WTP = willingness to pay.

**Figure A5.4** Point estimates and uncertainty of the around the benefits for the scenario and sensitivity analyses (PAS).


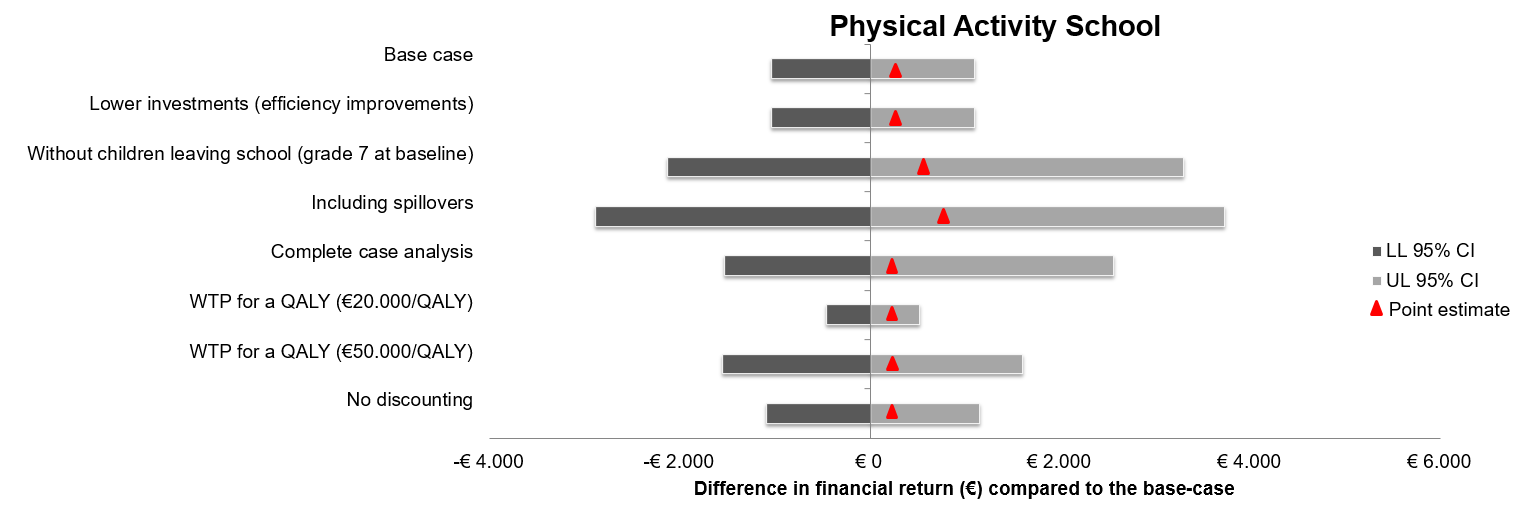


**Supplementary Figure A5.4** Notes: HRQOL = health-related quality of life; QALY = quality-adjusted life year; WTP = willingness to pay.

# Appendix 6. Quantitative outcomes not included in the SROI calculation

| ***PedsQL*** | | **Control schools** | **HPSF** | **PAS** | **Mean change HPSF vs. control** no adjustment | **Mean change HPSF vs. control** adjusted ^1^ | **Mean change PAS vs. control** no adjustment | **Mean change PAS vs. control**  adjusted ^1^ |
| --- | --- | --- | --- | --- | --- | --- | --- | --- |
|  |  | **Mean (SE) ^1^** | **Mean (SE) ^1^** | **Mean (SE) ^1^** |  |  |  |  |
| Total score, child-reported | T0 | 70.56 (0.6) | 71.13 (0.8) | 71.86 (0.8) |  |  |  |  |
|  | T1 | 76.72 (0.7) | 77.47 (0.7) | 77.80 (0.8) |  |  |  |  |
|  | T2 | 77.70 (0.7) | 77.37 (0.8) | 78.15 (0.8) | -3.60 (-12.8;5.6) | -3.42 (-12.8;5.9) | -3.37 (-12.6;5.9) | -3.5 (-12.7;5.6) |
| Total score, parent-reported | T0 | 84.08 (0.7) | 84.47 (0.5) | 85.11 (0.5) |  |  |  |  |
|  | T1 | 84.95 (0.6) | 84.95 (0.7) | 85.55 (0.7) |  |  |  |  |
|  | T2 | 85.43 (0.6) | 85.79 (0.6) | 85.94 (0.6) | -0.14 (-6.8;6.5) | 0.05 (-6.5;6.6) | -2.06 (-8.2;4.1) | -1.80 (-7.9;4.3) |
| Child- and parent-reported combined | T0 | 78.52 (0.6) | 79.09 (0.7) | 80.27 (0.7) |  |  |  |  |
|  | T1 | 80.20 (0.6) | 80.92 (0.6) | 82.00 (0.7) |  |  |  |  |
|  | T2 | 80.03 (0.6) | 79.79 (0.7) | 80.77 (0.7) | -0.81 (-2.3;1.4) | -0.66 (-2.8;1.5) | -1.02 (-3.1;1.1) | -0.63 (-2.7;1.4) |

**Table A6.1** Outcomes on children’s psychosocial health, post-imputation adjusted for covariates (PedsQL).

**Supplementary Figure A6.1**^1^ Mean difference adjusted for baseline values and covariates (sex, age, SES, ethnicity, BMI z-score at baseline).

Notes: HPSF = Healthy Primary School of the Future; PAS = Physical Activity School; PedsQL = Pediatric Quality of Life Inventory; SE= standard error.

**Table A6.2** Outcomes on parental absenteeism and parental time spent at unpaid work, post-imputation adjusted for covariates.

| **Outcome ^1^** | **Unit costs** | **Control schools**  %  (any absenteeism / time spent in unpaid work) | **HPSF**  %  (any absenteeism / time spent in unpaid work) | **PAS**  %  (any absenteeism / time spent in unpaid work) | **HPSF vs. control** | **PAS vs. control** |
| --- | --- | --- | --- | --- | --- | --- |
|  |  |  |  |  | Odds ratio ^3^ | Odds ratio ^3^ |
|  |  |  |  |  | Estimate (95% CI) | Estimate (95% CI) |
| Parental absenteeism from work due to sickness of the child | Paid work: €35.53 / hour ^2^ | 7.9% | 6.2% | 6.2% | 0.79 (0.29;2.12) | 0.79 (0.29;2.21) |
| Parental time spent at unpaid work/week | €14.31 / hour ^2^ | 8.7% | 7.7% | 7.5% | 0.89 (0.58;1.37) | 0.86 (0.56;1.30) |

**Supplementary Figure A6.2**^1^ Due to the small numbers (<10%), the potential benefits could not be reliably estimated and we refrained from including them in the calculation of financial return on investment.

^2^ Dutch guidelines for costing in health economic evaluations (7).

^3^ Ratio of any absenteeism/time spent in unpaid work for HPSF or PAS versus control schools.

Notes: HPSF = Healthy Primary School of the Future; PAS = Physical Activity School; SE= standard error.

# Appendix 7. Pre-imputation results

**Table A7.1** Benefits (€) (pre-imputation, adjusted for covariates).

| **Panel A: Net investments** | | | | | | | | | **HPSF versus control schools** | | | | |
| --- | --- | --- | --- | --- | --- | --- | --- | --- | --- | --- | --- | --- | --- |
|  |  |  |  |  |  |  |  |  | € per child (discounted results) | | | | |
| Net investment year 1 | |  |  |  |  |  |  |  | 440 |  |  | |  |
| Net investment year 2 | |  |  |  |  |  |  |  | 420 |  |  | |  |
| **Total net investments (year 1 and year 2) ^1^** | | | | | | | |  | **859** |  |  | |  |
| **Panel B: Benefits** | **Unit cost** | | **Control schools** | | **HPSF** |  | **PAS** |  | **HPSF versus control schools** | | | | |
|  |  | |  | |  |  |  |  | **Rate ratio ^2^** | | | **Benefits €** per child Y1+Y2 **^3^** | |
|  |  | | Mean | (SE) | Mean | (SE) | Mean | (SE) | Estimate | (95% CI) | | Estimate | (95% CI) |
| QALYs child | €36000 /QALY ^4^ | | 63769 | (412) | 68490 | (497) | 68598 | (505) | 0.98 | (0.94;1.02) | | -1214 | (-3642;1214) |
| Medical resource use | See Appendix 1^5^ | | -721 | (44) | -774 | (66) | -832 | (78) | 1.36 | (0.91;2.02) | | -247 | (-693;62) |
| HR absenteeism | €26.48 /day ^5,6^ | | -376 | (15) | -377 | (14) | -306 | (13) | 1.35 | (1.06;1.70) | | -125 | (-251;-21) |
| Other school absenteeism | €26.48 /day ^5,6^ | | -30 | (3) | -20 | (2) | -28 | (5) | 0.80 | (0.52;1.23) | | 5 | (-7;14) |
| **Total benefits(total year 1 and year 2)** | | | | | | | |  |  |  | | **-1581** | **(-4448;-596)** |
| **Panel C: Social return on investment** | | | | | | | | | **HPSF versus control schools** | | | | |
|  |  |  |  |  |  |  |  |  |  |  | **€** per child Y1+Y2 | | |
|  |  |  |  |  |  |  |  |  |  |  | Estimate | | (95% CI) |
| Ratio of benefits to investments ^7^ | | | | | | | |  |  |  | -1.8 | | (-4.2;0.3) |
| Net monetary benefit ^8^ | | | | | | | |  |  |  | -2440 | | (-4447;-596) |
| Net monetary benefit per child per day ^9^ | | | |  |  |  |  |  |  |  | -7.62 | | (-13.90;-1.86) |

| **Panel A: Net investments** | | | | | | | | | | | | | | | **PAS versus control schools** | | | | |
| --- | --- | --- | --- | --- | --- | --- | --- | --- | --- | --- | --- | --- | --- | --- | --- | --- | --- | --- | --- |
|  |  |  |  |  |  |  |  |  |  |  |  |  |  |  | € per child (discounted results) | | | | |
| Net investment year 1 | |  | |  | |  | |  | |  | |  |  | | 518 | |  |  |  |
| Net investment year 2 | |  | |  | |  | |  | |  | |  |  | | 499 | |  |  |  |
| **Total net investments (year 1 and year 2) ^1^** | | | | | | | | | | | | |  | | **1,017** | |  |  |  |
| **Panel B: Benefits** | **Unit cost** | | **Control schools** | | | | **HPSF** | |  | | **PAS** | |  | | **PAS versus control schools** | | | | |
|  |  | |  | | | |  | |  | |  | |  | | **Rate ratio ^2^** | | | **Benefits €** per child Y1+Y2 **^3^** | |
|  |  | | Mean | | (SE) | | Mean | | (SE) | | Mean | | (SE) | | Estimate | (95% CI) | | Estimate | (95% CI) |
| QALYs child | €36000 /QALY ^4^ | | 63769 | | (412) | | 68490 | | (497) | | 68598 | | (505) | | 1.01 | (0.97;1.05) | | 607 | (-1821;3035) |
| Medical resource use | See Appendix 1^5^ | | -721 | | (44) | | -774 | | (66) | | -832 | | (78) | | 1.08 | (0.73;1.59) | | -55 | (-405;185) |
| HR absenteeism | €26.48 /day ^5,6^ | | -376 | | (15) | | -377 | | (14) | | -306 | | (13) | | 0.95 | (0.74;1.18) | | 22 | (-64;93) |
| Other school absenteeism | €26.48 /day ^5,6^ | | -30 | | (3) | | -20 | | (2) | | -28 | | (5) | | 0.69 | (0.45;1.04) | | 9 | (-1;16) |
| **Total benefits(total year 1 and year 2)** | | | | | | | | | | | | |  | |  |  | | **582** | **(-1527;2564)** |
| **Panel C: Social return on investment** | | | | | | | | | | | | | | | **PAS versus control schools** | | | | |
|  |  |  |  |  |  |  |  |  |  |  |  |  |  |  |  | |  | **€** per child Y1+Y2 | |
|  |  |  |  |  |  |  |  |  |  |  |  |  |  |  |  | |  | Estimate | (95% CI) |
| Ratio of benefits to investments ^7^ | | | | | | | | | | | | | |  |  | |  | 0.6 | (-1.5;2.5) |
| Net monetary benefit ^8^ | | | | | | | | | | | | | |  |  | |  | -435 | (-2544;1547) |
| Net monetary benefit per child per day ^9^ | | | | | |  | |  | |  | |  | |  |  | |  | -1.36 | (-7.95;4.84) |

**Table A7.1**[continued]

**Supplementary Figure A7.1** ^1^ Net investment = investments minus delivery-related offsets (HPSF: household expenses on lunches for children, and the value of the extended school day for parental productivity). ^2^ Ratio of mean benefits for HPSF or PAS versus control schools.
^3^ Benefits for HPSF or PAS = mean value at control schools * rate ratio (repeated for lower and upper bound of the confidence interval). Discounted with an annual discount rate of2.5% to account for differential timing of investments and benefits.
^4^ Pomp, M., Schoemaker, C.G., Polder, J.J. (2014). Op weg naar maatschappelijke kosten-batenanalyses voor preventie en zorg [Social cost-benefit analysis for prevention and care]. Ministerie van Volksgezondheid. Available online at: https://www.rivm.nl/publicaties/op-weg-naar-maatschappelijke-kosten-batenanalyses-voor-preventie-en-zorg-themarapport. [Accessed March 1, 2019]. (5).

5 Because medical resource use and school absenteeism represent a cost, they are represented as a negative financial return.

^6^ Drost, R., Paulus, A., Ruwaard, D., Evers, S. (2014). Handleiding intersectorale kosten en baten van (preventieve) interventies [Guideline for intersectoral costs and benefits of preventive interventions]. Available online at: https://hsr.mumc.maastrichtuniversity.nl/sites/intranet.mumc.maastrichtuniversity.nl/files/hsr_mumc_maastrichtuniversity_nl/Symposia/30_Oct_2014_VGE_NVTAG/um-hsr_handleiding_intersectorale_kosten_en_baten.pdf. [Accessed November 25, 2019]. (6).
^7^ Ratio of total of benefits and net investments. ^8^ Incremental net monetary benefit = incremental benefit – incremental net investment.
^9^ For a total of 160 schooldays per year (total of 320 days for two years).
Notes: CI = confidence interval; HPSF = Healthy Primary School of the Future; HR = health-related; IQR = interquartile range; PAS = Physical Activity School; QALYs = quality-adjusted life years; SE= standard error.

**Table A7.2** Benefits and net investments per child (€) in year 1 and year 2 (post-imputation, not adjusted for covariates).

| **Panel A: Net investments** | | | | | | | | | | | **HPSF versus control schools** | | | | | | | |
| --- | --- | --- | --- | --- | --- | --- | --- | --- | --- | --- | --- | --- | --- | --- | --- | --- | --- | --- |
|  |  |  |  |  |  |  |  |  |  |  | € per child (discounted results) | | | | | | | |
| Net investment year 1 |  |  |  |  | |  |  | | |  | 440 | |  | |  | | |  |
| Net investment year 2 |  |  |  |  | |  |  | | |  | 420 | |  | |  | | |  |
| **Total social opportunity costs (year 1 and year 2) ^1^** | | | | | | | | | |  | **859** | |  | |  | | |  |
| **Panel B: Benefits** | **Unit cost** | **Control schools** | | | **HPSF** |  | | **PAS** | |  | **HPSF versus control schools** | | | | | | | |
|  |  |  | | |  |  | |  | |  | **Rate ratio ^2^** | | | | | | **Benefits €** per child Y1+Y2 **^3^** | |
|  |  | Mean | (SE) | | Mean | (SE) | | Mean | | (SE) | Estimate | | | | | (95% CI) | Estimate | (95% CI) |
| QALYs child | €36000 /QALY ^4^ | 68508 | (335.0) | | 68554 | (332.6) | | 68531 | | (319.6) | 1.00 | | | | | (0.98;1.02) | 0 | (-1304;1304) |
| Medical resource use | See Appendix 1^5^ | -1056 | (211.3) | | -997 | (204.9) | | -1010 | | (194.4) | 0.97 | | | | | (0.80;1.17) | 30 | (-171;120) |
| HR absenteeism | €26.48 /day ^5,6^ | -338 | (15.6) | | -352 | (17.6) | | -309 | | (15.9) | 1.05 | | | | | (0.92;1.19) | -16 | (-61;26) |
| Other school absenteeism | €26.48 /day ^5,6^ | -31 | (6.8) | | -25 | (7.4) | | -29 | | (6.6) | 0.81 | | | | | (0.57;1.15) | 6 | (-4;13) |
| **Total benefits (total year 1 and year 2)** | | | | | | | | | |  |  | | | | |  | **20** | **(-2933;3673)** |
| **Panel C: Social return on investment** | | | | | | | | | | | **HPSF versus control schools** | | | | | | | |
|  |  |  |  |  |  |  |  |  |  |  |  |  | | **€** per child Y1+Y2 | | | | |
|  |  |  |  |  |  |  |  |  |  |  |  |  | | Estimate | | | (95% CI) | |
| Ratio of benefits to investments ^7^ | | | | | | | | |  | |  |  | | 0.02 | | | (-1.2;1.2) | |
| Net monetary benefit ^8^ | | | | | | | | |  | |  |  | | -840 | | | (-1924;208) | |
| Net monetary benefit per child per day ^9^ | | |  |  | |  |  | |  | |  |  | | -2.62 | | | (-6.01;0.65) | |

| **Panel A: Net investments** | | | | | | | | | | | | | | | **PAS versus control schools** | | | | | |
| --- | --- | --- | --- | --- | --- | --- | --- | --- | --- | --- | --- | --- | --- | --- | --- | --- | --- | --- | --- | --- |
|  |  |  |  |  |  |  |  |  |  |  |  |  |  |  | € per child (discounted results) | | | | | |
| Net investment year 1 | |  | |  | |  | |  | |  | |  |  | | 518 |  |  | | |  |
| Net investment year 2 | |  | |  | |  | |  | |  | |  |  | | 499 |  |  | | |  |
| **Total social opportunity costs (year 1 and year 2) ^1^** | | | | | | | | | | | | |  | | **1,017** |  |  | | |  |
| **Panel B: Benefits** | **Unit cost** | | **Control schools** | | | | **HPSF** | |  | | **PAS** | |  | | **PAS versus control schools** | | | | | |
|  |  | |  | | | |  | |  | |  | |  | | **Rate ratio ^2^** | | | **Benefits €** per child Y1+Y2 **^3^** | | |
|  |  | | Mean | | (SE) | | Mean | | (SE) | | Mean | | (SE) | | Estimate | (95% CI) | | Estimate | (95% CI) | |
| QALYs child | €36000 /QALY ^4^ | | 68508 | | (335.0) | | 68554 | | (332.6) | | 68531 | | (319.6) | | 1.00 | (0.98;1.02) | | 0 | (-1304;1304) | |
| Medical resource use | See Appendix 1^5^ | | -1056 | | (211.3) | | -997 | | (204.9) | | -1010 | | (194.4) | | 0.97 | (0.80;1.16) | | 30 | (-161;201) | |
| HR absenteeism | €26.48 /day ^5,6^ | | -338 | | (15.6) | | -352 | | (17.6) | | -309 | | (15.9) | | 0.91 | (0.80;1.05) | | 29 | (-16;64) | |
| Other school absenteeism | €26.48 /day ^5,6^ | | -31 | | (6.8) | | -25 | | (7.4) | | -29 | | (6.6) | | 0.90 | (0.65;1.25) | | 3 | (-7;10) | |
| **Total benefits (total year 1 and year 2)** | | | | | | | | | | | | |  | |  |  | | **62** | **(-2050;98)** | |
| **Panel C: Social return on investment** | | | | | | | | | | | | | | | **PAS versus control schools** | | | | | |
|  |  |  |  |  |  |  |  |  |  |  |  |  |  |  |  |  | **€** per child Y1+Y2 | | | |
|  |  |  |  |  |  |  |  |  |  |  |  |  |  |  |  |  | Estimate | | (95% CI) | |
| Ratio of benefits to investments ^7^ | | | | | | | | | | | | | |  |  |  | 0.06 | | (-1.0;1.1) | |
| Net monetary benefit ^8^ | | | | | | | | | | | | | |  |  |  | -955 | | (-2046;103) | |
| Net monetary benefit per child per day ^9^ | | | | | |  | |  | |  | |  | |  |  |  | -2.98 | | (-6.39;0.32) | |

**Table A7.2** [continued]

**Supplementary Figure A7.2** ^1^ Net investment = investments minus delivery-related offsets (HPSF: household expenses on lunches for children, and the value of the extended school day for parental productivity). ^2^ Ratio of mean benefits for HPSF or PAS versus control schools.
^3^ Benefits for HPSF or PAS = mean value at control schools * rate ratio (repeated for lower and upper bound of the confidence interval). Discounted with an annual discount rate of2.5% to account for differential timing of investments and benefits.
^4^ Pomp, M., Schoemaker, C.G., Polder, J.J. (2014). Op weg naar maatschappelijke kosten-batenanalyses voor preventie en zorg [Social cost-benefit analysis for prevention and care]. Ministerie van Volksgezondheid. Available online at: https://www.rivm.nl/publicaties/op-weg-naar-maatschappelijke-kosten-batenanalyses-voor-preventie-en-zorg-themarapport. [Accessed March 1, 2019]. (5).
^5^ Because medical resource use and school absenteeism represent a cost, they are represented as a negative financial return.

^6^ Drost, R., Paulus, A., Ruwaard, D., Evers, S. (2014). Handleiding intersectorale kosten en baten van (preventieve) interventies [Guideline for intersectoral costs and benefits of preventive interventions]. Available online at: https://hsr.mumc.maastrichtuniversity.nl/sites/intranet.mumc.maastrichtuniversity.nl/files/hsr_mumc_maastrichtuniversity_nl/Symposia/30_Oct_2014_VGE_NVTAG/um-hsr_handleiding_intersectorale_kosten_en_baten.pdf. [Accessed November 25, 2019]. (6).

^7^ Ratio of total of benefits and net investments. ^8^ Incremental net monetary benefit = incremental benefit – incremental net investment.
^9^ For a total of 160 schooldays per year (total of 320 days for two years).
Notes: CI = confidence interval; HPSF = Healthy Primary School of the Future; HR = health-related; IQR = interquartile range; PAS = Physical Activity School; QALYs = quality-adjusted life years; SE= standard error.

# References

1. Oosterhoff, M., Bosma, H., van Schayck, O.C.P., Joore, M.A. (2018). *A Cost Analysis of School-Based Lifestyle Interventions.* Prev Sci. 19(6):716-27. doi: 10.1007/s11121-018-0918-1.
2. Willeboordse, M., Jansen, M.W., van den Heijkant, S.N., Simons, A., Winkens, B., de Groot, R.H., et al. (2016). *The Healthy Primary School of the Future: study protocol of a quasi-experimental study.* BMC public health. 16:639. doi: 10.1186/s12889-016-3301-9.
3. Bartelink, N.H.M., van Assema, P., Kremers, S.P.J., Savelberg, H.H., Oosterhoff, M., Willeboordse, M., et al. (2019). *One- and Two-Year Effects of the Healthy Primary School of the Future on Children's Dietary and Physical Activity Behaviours: A Quasi-Experimental Study.* Nutrients. 11(3). doi: 10.3390/nu11030689.
4. Bartelink, N., Van Assema, P., Kremers, S.P.J., Savelberg, H.H., Oosterhoff, M., Willeboordse, M., et al. (2019). *Can the Healthy Primary School of the Future offer perspective in the on-going obesity epidemic in young children? – a quasi-experimental study.* BMJ Open. 9(10): e030676. doi: 10.1136/bmjopen-2019-030676.
5. Pomp, M., Schoemaker, C.G., Polder, J.J. (2014). *Op weg naar maatschappelijke kosten-batenanalyses voor preventie en zorg [Social cost-benefit analysis for prevention and care].* Ministerie van Volksgezondheid. Available online at: https://www.rivm.nl/publicaties/op-weg-naar-maatschappelijke-kosten-batenanalyses-voor-preventie-en-zorg-themarapport. [Accessed March 1, 2019].
6. Drost, R., Paulus, A., Ruwaard, D., Evers, S. (2014). *Handleiding intersectorale kosten en baten van (preventieve) interventies [Guideline for intersectoral costs and benefits of preventive interventions]*. Available online at: https://hsr.mumc.maastrichtuniversity.nl/sites/intranet.mumc.maastrichtuniversity.nl/files/hsr_mumc_maastrichtuniversity_nl/Symposia/30_Oct_2014_VGE_NVTAG/um-hsr_handleiding_intersectorale_kosten_en_baten.pdf. [Accessed November 25, 2019].
7. Zorginstituut Nederland. (2015). *Kostenhandleiding: Methodologie van kostenonderzoek en referentieprijzen voor economische evaluaties in de gezondheidszorg [Methodology of cost research and cost prices for heal economic evaluations].* Available online at: https://www.zorginstituutnederland.nl/over-ons/werkwijzen-en-procedures/adviseren-over-en-verduidelijken-van-het-basispakket-aan-zorg/beoordeling-van-geneesmiddelen/richtlijnen-voor-economische-evaluatie. [Accessed November 14, 2019].
8. Lamers, L.M., Stalmeier, P.F., McDonnell, J., Krabbe, P.F., van Busschbach, J.J. (2005). *[Measuring the quality of life in economic evaluations: the Dutch EQ-5D tariff].* NTvG. 149(28):1574-8.
9. KPMG. (2014). *Inzicht in tarieven WMO en jeugdzorg Fase 2. [Tariffs for WMO and Youth Care].* Available online at: https://vng.nl//files/vng/201404_eindrapportage_inzicht_in_tarieven_wmo_en_jeugdzorg.pdf [Accessed August 12, 2019].
10. Shavers, V.L. (2007). *Measurement of socioeconomic status in health disparities research.* J Natl Med Assoc. 99(9):1013.
11. Keij, I. (2000). *Hoe doet het CBS dat nou? Standaarddefinitie allochtonen [Definition immigrants].* Index: Feiten en Cijfers over onze Samenleving. 10:24-5.Available online at:

https://www.cbs.nl › media › imported › documents › index1119. [Accessed November 13, 2019].

1. Schönbeck, Y., Talma, H., van Dommelen, P., Bakker, B., Buitendijk, S.E., Hirasing, R.A., et al. (2011). *Increase in Prevalence of Overweight in Dutch Children and Adolescents: A Comparison of Nationwide Growth Studies in 1980, 1997 and 2009.* PLoS ONE. 6(11):e27608. doi: 10.1371/journal.pone.0027608.
2. Oosterhoff, M., Bosma, H., van Schayck, O.C.P., Joore, M.A. (2019). *Correction to: Cost Analysis of School-Based Lifestyle Interventions.* Prev Sci. doi: 10.1007/s11121-019-01030-4.
3. Fornari, L.S., Giuliano, I., Azevedo, F., Pastana, A., Vieira, C., Caramelli, B. (2013). *Children First Study: how an educational program in cardiovascular prevention at school can improve parents' cardiovascular risk.* Eur J Prev Cardiol. 20(2):301-9. doi: 10.1177/2047487312437617.
4. Gunawardena, N., Kurotani, K., Indrawansa, S., Nonaka, D., Mizoue, T., Samarasinghe, D. (2016). *School-based intervention to enable school children to act as change agents on weight, physical activity and diet of their mothers: a cluster randomized controlled trial.* Int J Behav Nutr Phys Act. 2016;13:45. doi: 10.1186/s12966-016-0369-7.
5. van den Berg, M., De Wit, G.A., Vijgen, S.M.C., Busch, M.C.M., Schuit, A.J. (2008). *Kosteneffectiviteit van preventie: kansen voor het Nederlandse volksgezondheidsbeleid [Cost-effectiveness of prevention].* NTvG. 152:1329-34.
